# Supplementary material for: Essential dynamic interdependence of FtsZ and SepF for Z-ring and septum formation in Corynebacterium glutamicum
Source: Nat Commun. 2020 Apr 2;11:1641. doi: 10.1038/s41467-020-15490-8 (PMC7118173; doi:10.1038/s41467-020-15490-8)
Supplement: Supplementary file 1 — Supplementary Information [file 41467_2020_15490_MOESM1_ESM.pdf]

Supplementary Information

Essential dynamic interdependence of FtsZ and SepF for Z-ring and septum formation in *Corynebacterium glutamicum*

Sogues, et al

Supplementary Figures 1-26

Supplementary Tables 1-6

Supplementary References

Supplementary Figures

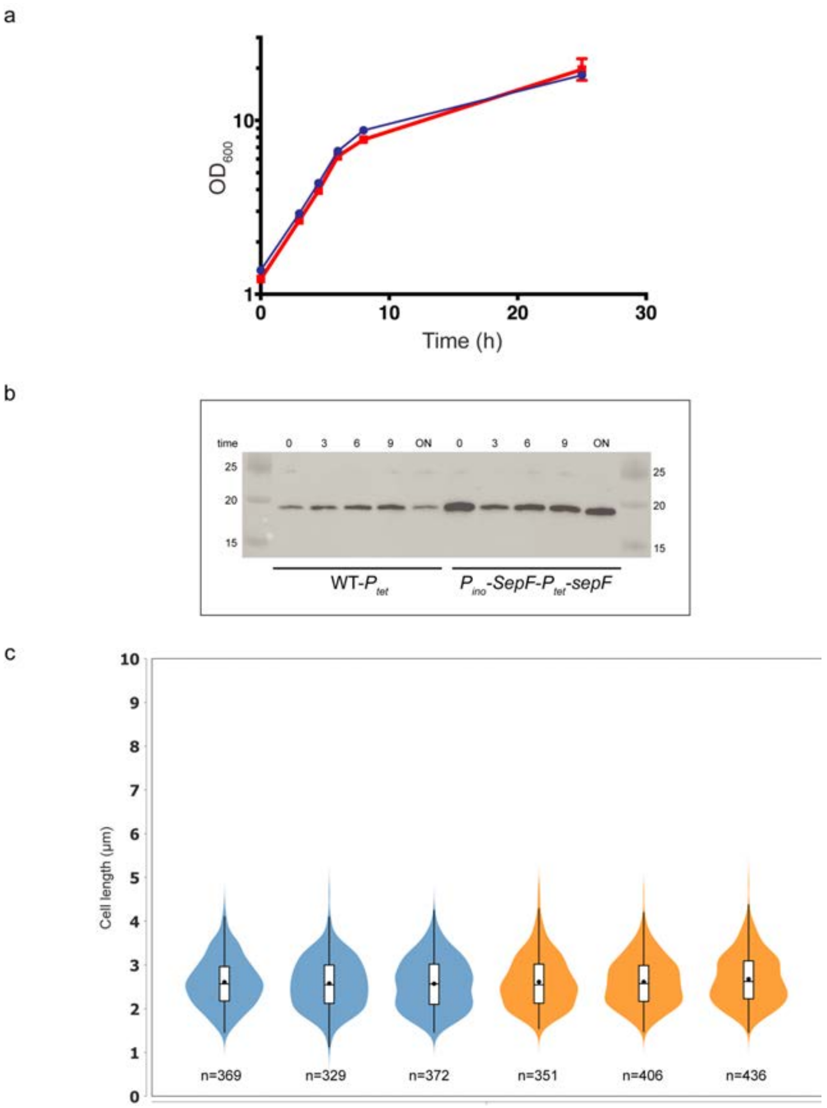

Supplementary Figure 1: Complementation of the P_{ino}-*sepF* strain during SepF depletion by the expression of P_{tet}-*sepF* upon tetracycline addition. a. Growth curves comparing WT-P_{tet} (blue) with P_{ino}-*sepF*-P_{tet}-*sepF* (red) in 1% *myo*-inositol + 50 ng/ml tetracycline. Error bars represent the mean ± SD. **b.** Western blot of whole cell extracts corresponding to the above growth curve at different time points (0h, 3h, 6h, 9h, and over-night (ON)) probed with anti-SepF antibodies to confirm SepF expression during complementation of the SepF depleted strain. Molecular weight markers (kDa) are shown on each side of the blot. The blot shown is representative of experiments made independently in triplicate. **c.** Violin plot of triplicate analysis showing the distribution of cell length at time point 4.5 hours after *myo*-inositol and tetracycline addition for WT-P_{tet} (blue) and P_{ino}-*sepF*-P_{tet}-*sepF* (orange). Mean values and standard deviations of cell lengths are shown in Supplementary Table 5. The number of cells used in the analyses (n) is indicated below each violin representation. The box indicates the 25th to the 75th percentile and the whiskers indicate the 95% confidence interval. The mean and the median are indicated with a dot and a line in the box, respectively. Source data are provided as a Source Data file.

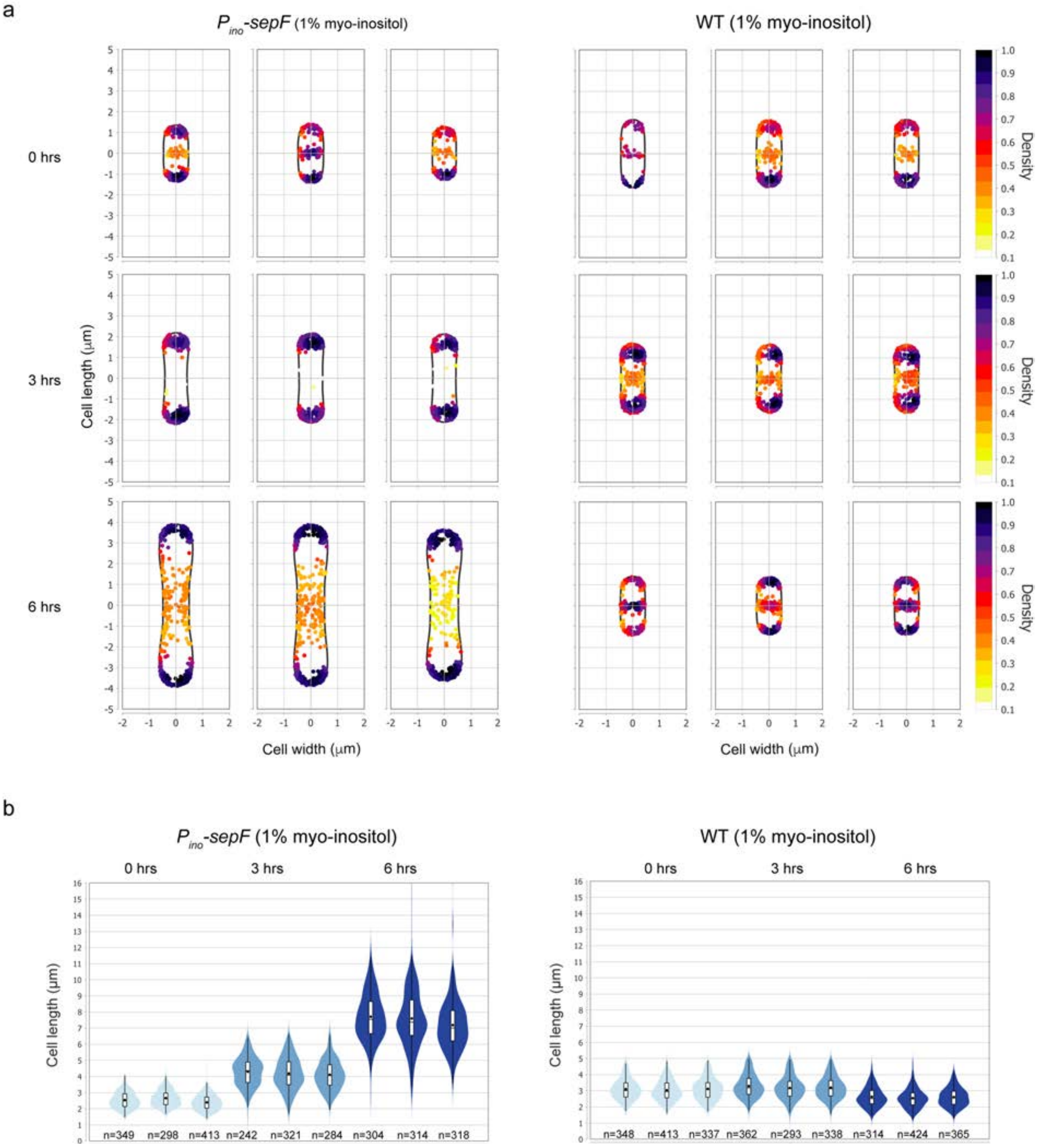

Supplementary Figure 2: Triplicate analysis of fluorescent HADA localization and cell length during SepF depletion. a. Heat maps representing the localization pattern of HADA at 0, 3 and 6 hours after *myo*-inositol addition for P_{ino}-*sepF* (left group of triplicates) and WT (right group of triplicates) strains. **b.** Violin plot of triplicate analysis showing the distribution of cell length at time points 0, 3, 6 hours after *myo*-inositol addition for P_{ino}-*sepF* and WT. Mean values and standard deviations of cell lengths are shown in Supplementary Table 5. Mean values between triplicate time points are statistically different (two-tailed p-values derived from a Mann-Whitney test are shown in Supplementary Table 6) for P_{ino}-*sepF*. The number of cells used in the analyses (n) is indicated below each violin representation. The box indicates the 25th to the 75th percentile and the whiskers indicate the 95% confidence interval. The mean and the median are indicated with a dot and a line in the box, respectively. Source data are provided as a Source Data file.

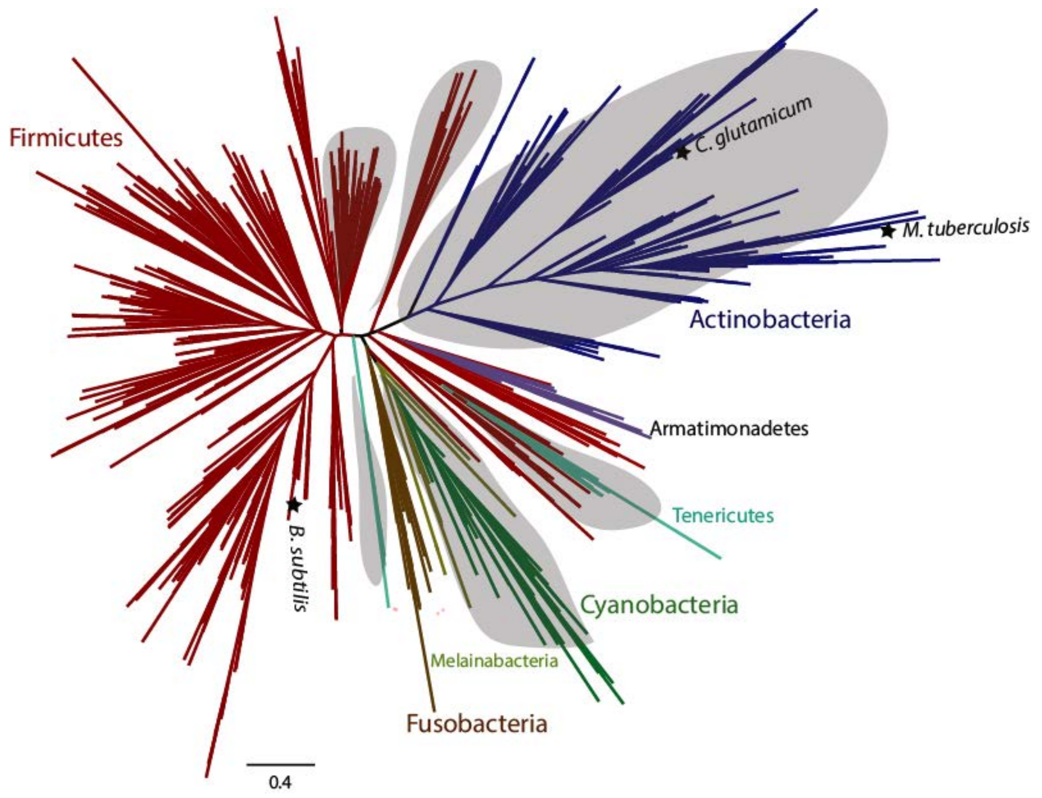

Supplementary Figure 3: Maximum likelihood phylogenetic tree of bacterial SepF proteins. Major Phyla are shown in different colors. Grey-shaded areas highlight branches with no detectable *ftsA* homologue, as is the case for *Actinobacteria*, *Cyanobacteria*, *Melainabacteria*, *Tenericutes* and some clades within *Firmicutes*. The scale bar represents average substitutions per site.

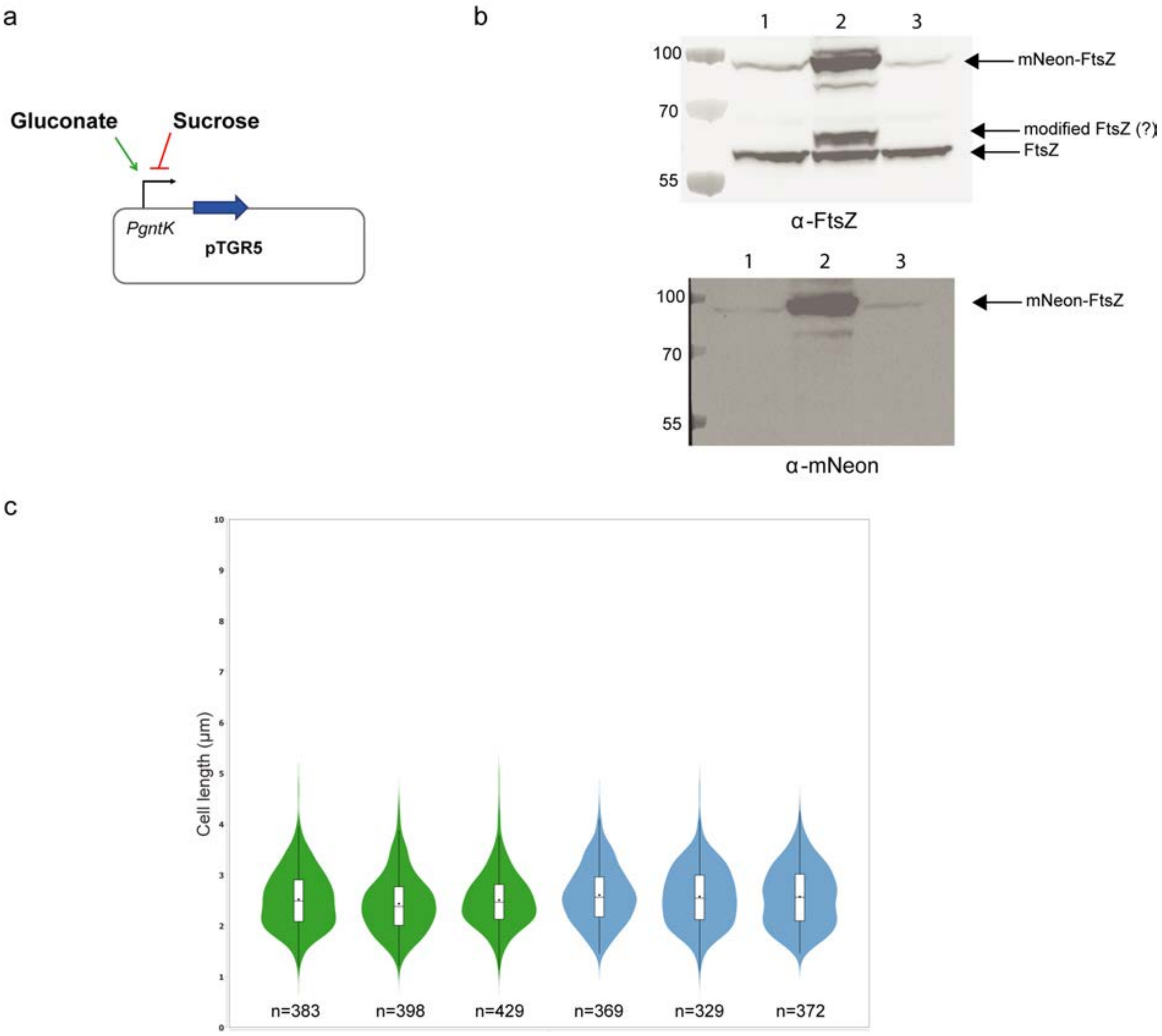

Supplementary Figure 4: mNeon-FtsZ expression system: **a.** Schematic representation of the P_{gntK} promotor in the pTGR5 vector backbone. The P_{gntK} promotor is repressed in the presence of 4% sucrose and maximally induced by 1% gluconate. **b.** Western blot of whole cell extracts of WT-P_{gntK}-*mneon-ftsZ* cells grown in either 4% sucrose (Lane 1) or 4% sucrose + 1% gluconate (Lane 2) or 4% sucrose + 1% myo-inositol (Lane 3) to show that myo-inositol does not affect FtsZ levels. The blot on top was revealed with an anti-FtsZ antibody, the blot on the bottom with anti-mNeon antibody. Molecular weight markers (kDa) are shown on the left side of the blot. The blots shown are representative of experiments made independently in triplicate. **c.** Comparison of the size distribution in exponential phase of WT-P_{gntK} (blue) and WT-P_{gntK}-*mneon-ftsZ* (green). Mean values and standard deviations of cell lengths are shown in Supplementary Table 5. The number of cells used in the analyses (n) is indicated below each violin representation. The box indicates the 25th to the 75th percentile and the whiskers indicate the 95% confidence interval. The mean and the median are indicated with a dot and a line in the box, respectively. Source data are provided as a Source Data file.

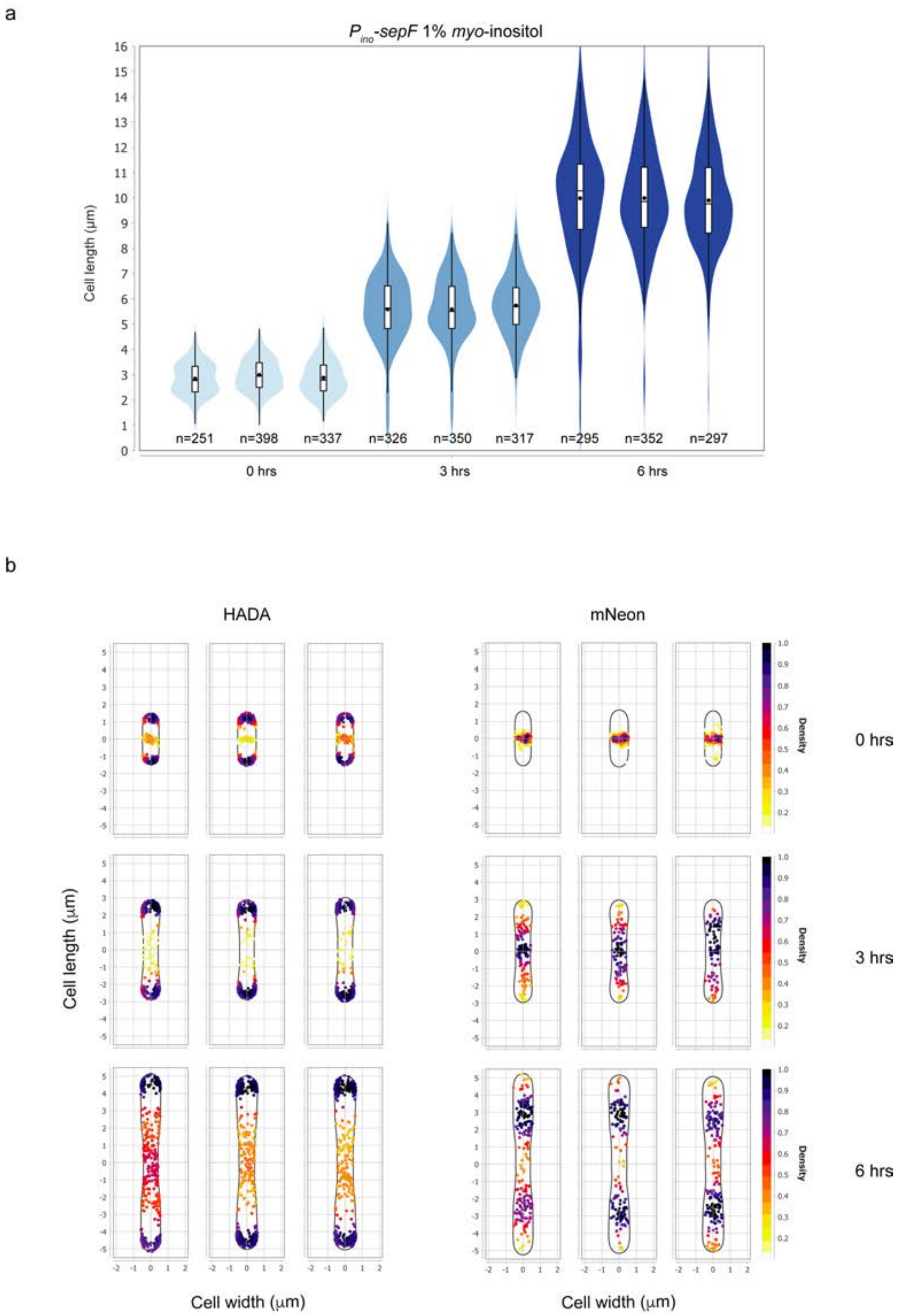

Supplementary Figure 5: Characterization of the *P*$_{ino}$*-sepF-P*$_{gntK}$*-mneon-FtsZ* strain.
a. Violin plot of triplicate analysis showing the cell length distribution at time points 0, 3, 6 hours after *myo*-inositol addition for cells grown in minimal medium supplemented with 4% sucrose. Mean values of triplicates were identical at each time point (Mann-Whitney) and mean values of two different time points for one sample were different (two-tailed p-values derived from a Mann-Whitney test are shown in Supplementary Table 6). The number of cells used in the analyses (n) is indicated below each violin representation. The box indicates the 25th to the

75th percentile and the whiskers indicate the 95% confidence interval. The mean and the median are indicated with a dot and a line in the box, respectively. **b.** Triplicate heatmaps representing the localization pattern of HADA (left) and mNeon-FtsZ (right) for the above strain at 0, 3 and 6 hours. Mean values and standard deviations of cell lengths are shown in Supplementary Table 5. Source data are provided as a Source Data file.

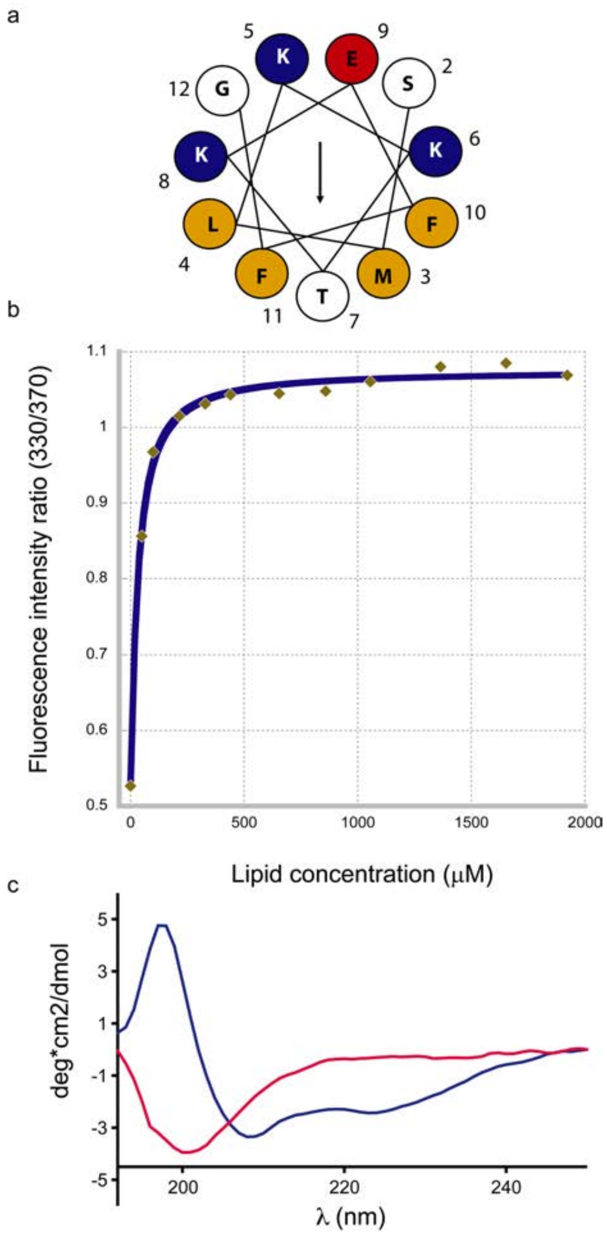

Supplementary Figure 6. SepF$_M$ peptide-membrane interactions. a. Amphipathic helix prediction of the membrane-binding region of SepF (SepF$_M$). **b.** Tryptophan fluorescence titration assay using a modified SepF$_M$ peptide (including a C-terminal tryptophan residue) as a function of lipid concentration (see Supplementary Methods for details). **c.** Circular dichroism spectra of SepF$_M$ in the absence (red) and presence (blue) of SUVs.

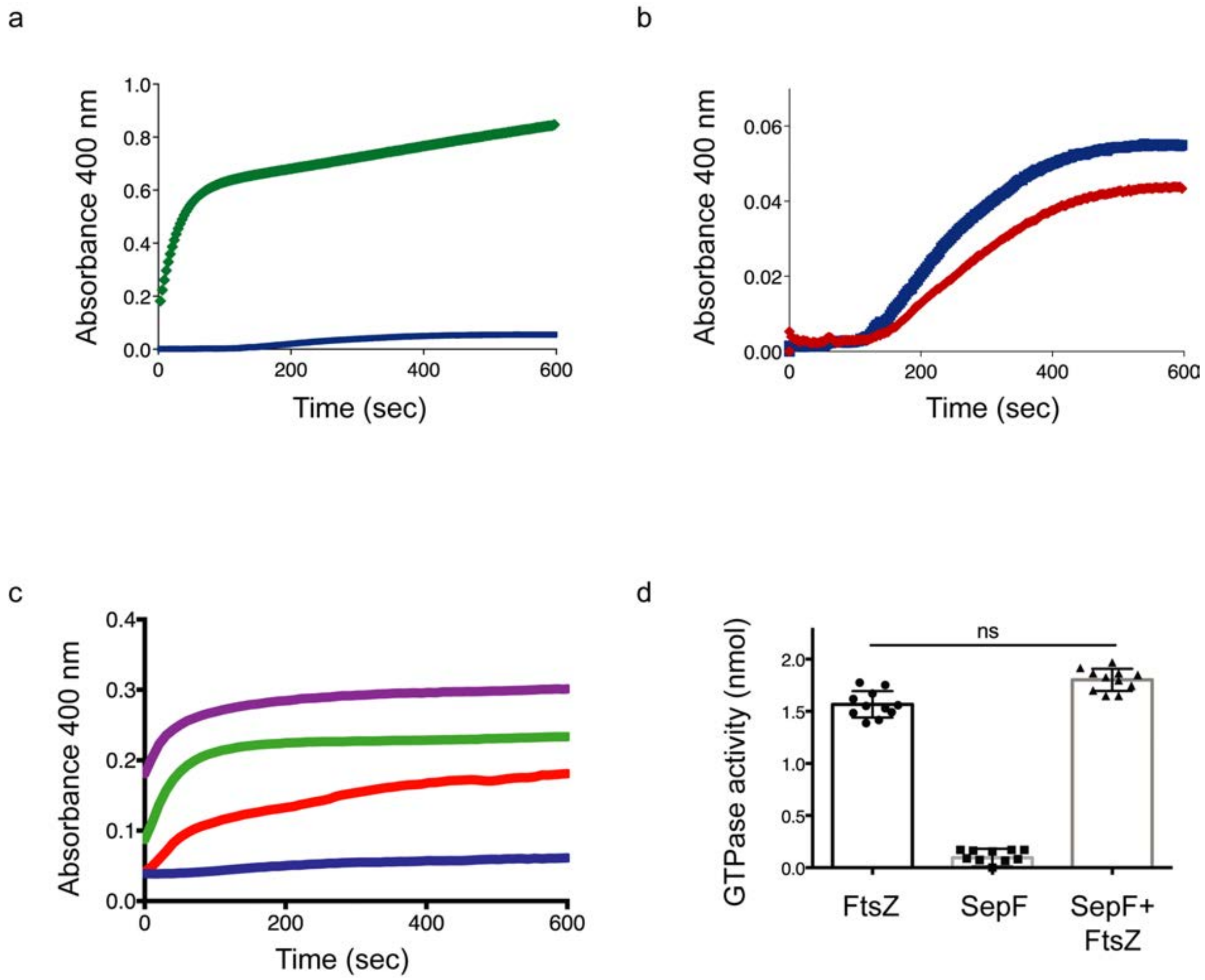

Supplementary Figure 7: FtsZ polymerization assays in the presence of SepF mutants. **a.** The absorbance measured at 400 nm for FtsZ alone (15 μM, blue curve) and FtsZ plus $SepF_{\Delta ML}$ (15 μM each, green curve). **b.** The absorbance measured at 400 nm for FtsZ alone (15 μM, blue curve) and FtsZ plus $SepF_{K125E/F131A}$ (15 μM each, red curve). **c.** The absorbance measured at 400 nm for FtsZ (15 μM) in the presence of GTP (0.5 mM, blue curve) and GMPCPP (0.5 mM, red curve) or FtsZ plus SepF (15 μM each) in the presence of GTP (0.5 mM, green curve) and GMPCPP (0.5 mM, purple curve). **d**. GTPase activity (nmol of phosphate release) of 15 μM SepF and 15 μM FtsZ in the absence or presence of SepF (15 μM). The differences between FtsZ in the presence or absence of SepF are not statistically significant (F test, p=0.5872). Error bars represent the mean ± SD. n=11 independent measurements. Mean value is 1.56 for FtsZ, 0.09 for SepF and 1.80 for SepF+FtsZ. Source data are provided as a Source Data file.

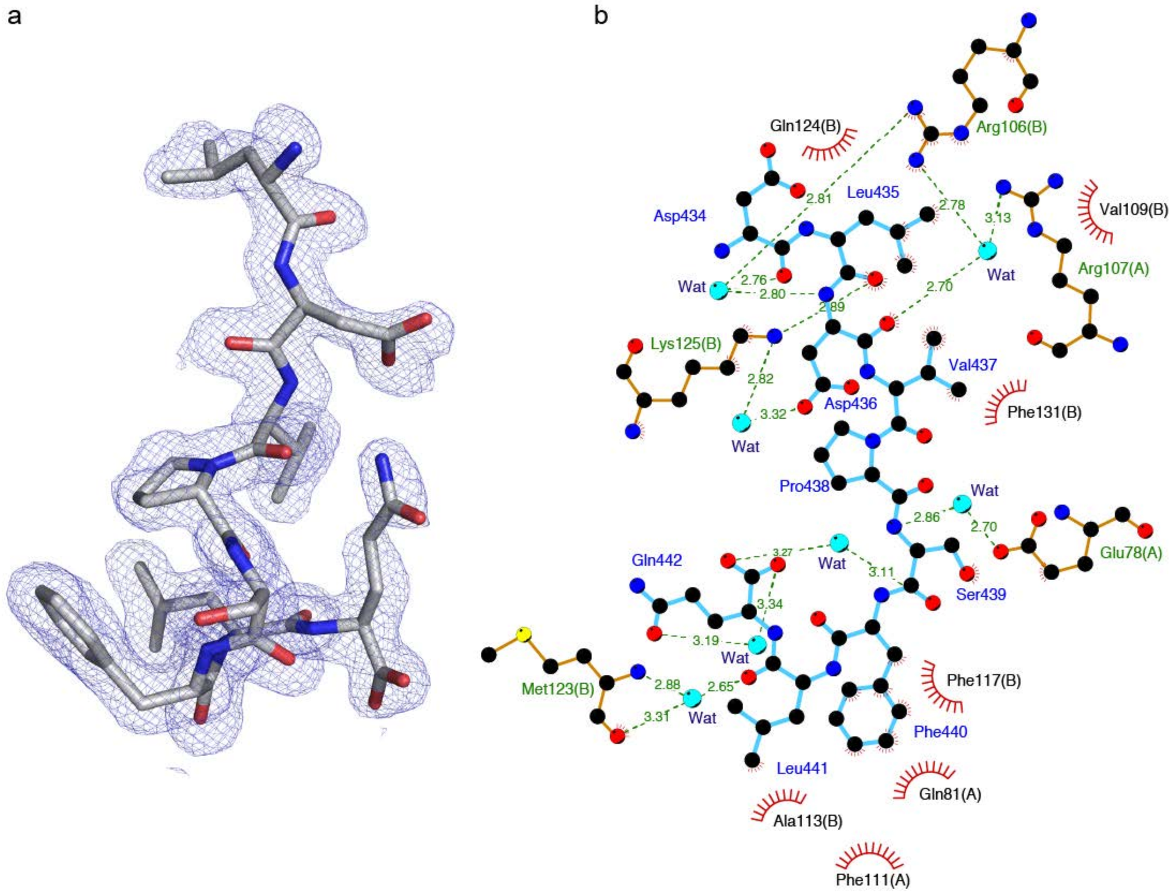

Supplementary Figure 8: Intermolecular interactions in the SepF-FtsZ$_{CTD}$ complex. a. Final 2Fo-Fc electron density map of FtsZ$_{CTD}$ (contoured at 1.1 σ). **b.** Interactions made by peptide FtsZ$_{CTD}$ (blue) with residues in the SepF binding pocket. The plot was made with LIGPLOT (Supplementary Ref. 1).

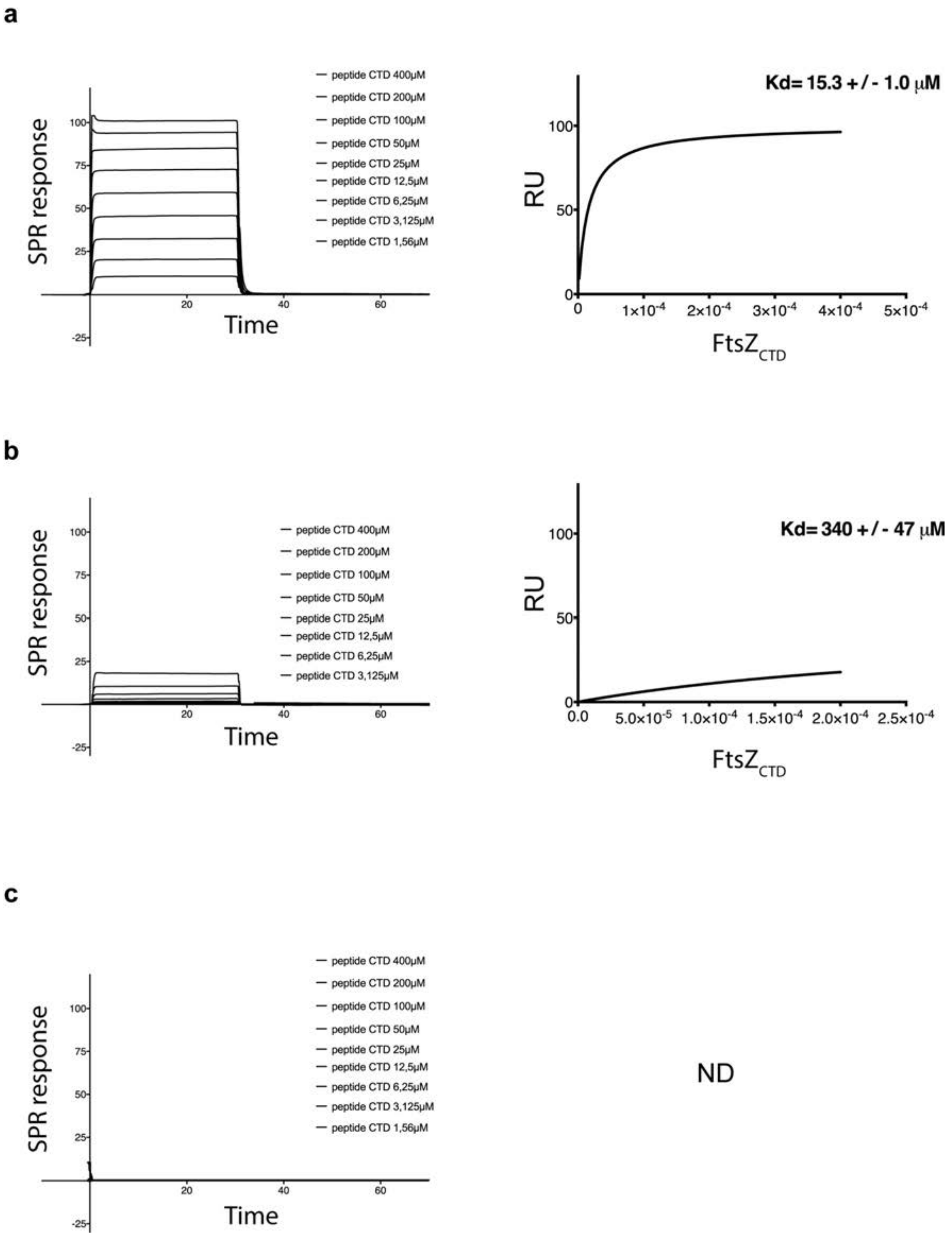

Supplementary Figure 9: SPR binding profiles and fitted curves for $FtsZ_{CTD}$ interactions with: (a) $SepF_{\Delta ML}$, (b) $SepF_{\Delta ML,F131A}$ and (c) $SepF_{\Delta ML,K125/F131A}$.

a

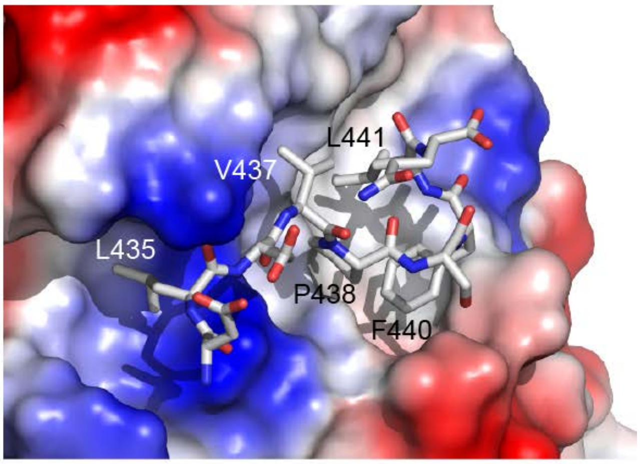

b

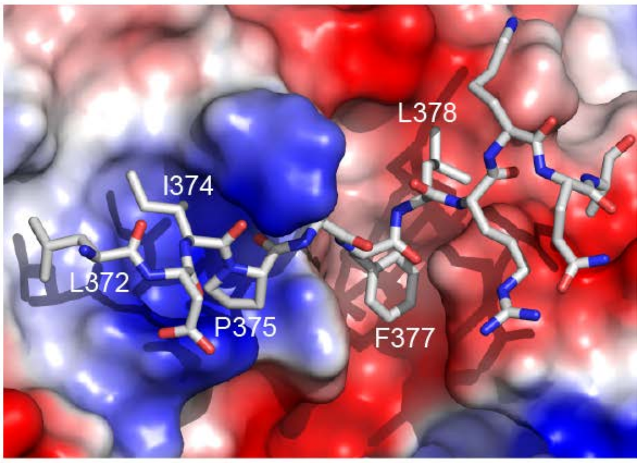

c

E. coli FtsZ	371	**YLD**I**PAFL**R	379
C. glut FtsZ	434	D**LDVP**S**FL**Q	442

Supplementary Figure 10: The same conserved hydrophobic residues of FtsZ$_{CTD}$ are involved in protein contacts with SepF and SlmA. a. Structure of the FtsZ$_{CTD}$ peptide (in stick representation) bound to the *C. glutamicum* SepF binding pocket color-coded according to surface electrostatic potential. **b.** Idem for FtsZ$_{CTD}$ bound to *E. coli* SlmA. **c.** Partial sequence alignment of FtsZ$_{CTD}$ from *C. glutamicum* and *E. coli*. Hydrophobic residues in bold are labelled in **a** and **b**.

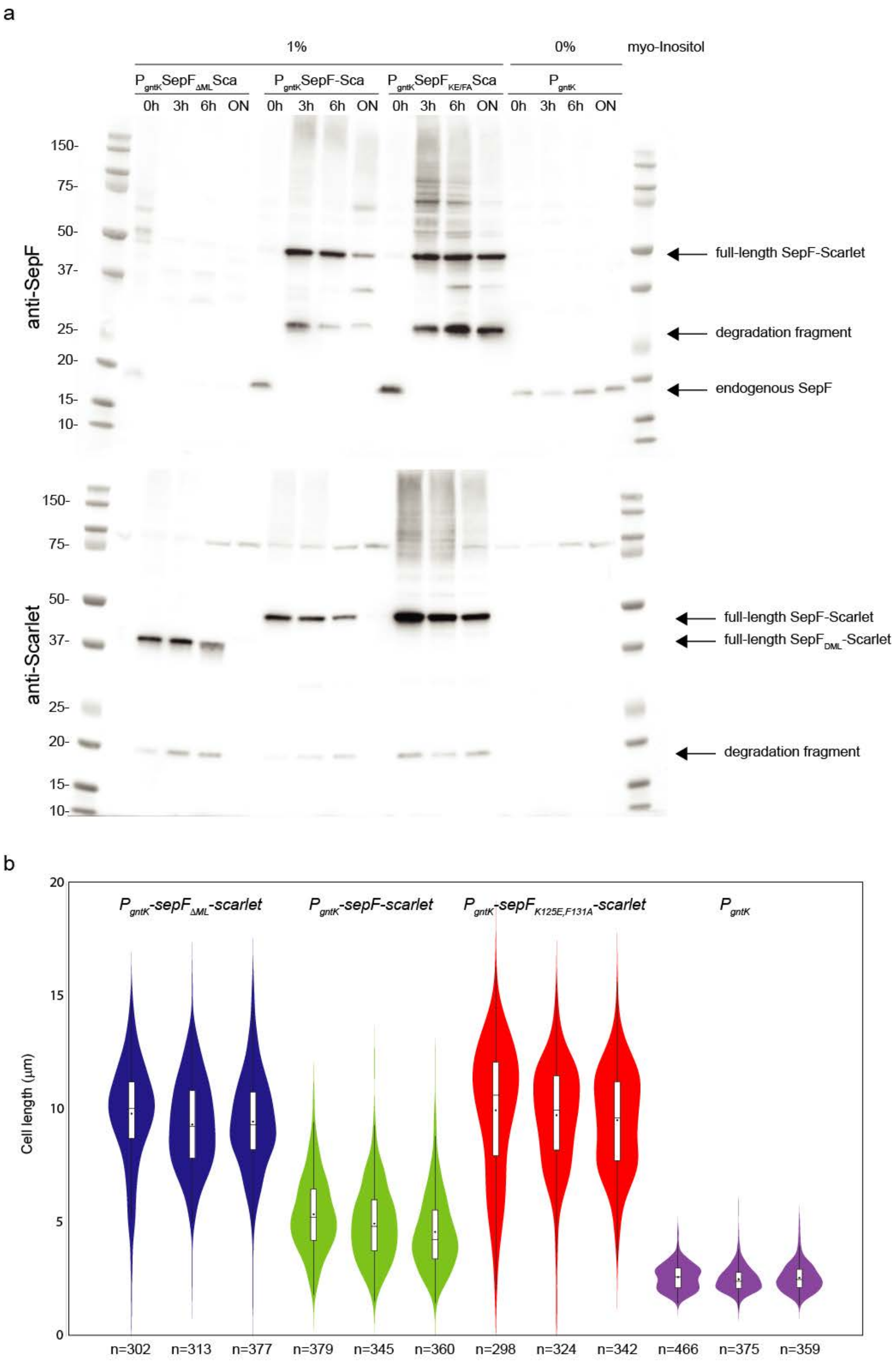

Supplementary Figure 11: Complementation of the *P_{ino}-sepF* strain by the expression of *P_{gntK}-sepF-scarlet* mutants. a. Western blot of whole cell extracts corresponding to the growth curve shown in Fig. 3 at different time points (0h, 3h, 6h, ON) probed with anti-SepF and anti-Scarlet antibodies to confirm protein expression. Molecular weight markers (kDa) are shown on each side of the blot. The blots shown are representative of experiments made independently in triplicate. **b.** Violin plot of triplicate analysis showing the cell length distribution at 6 hours for the *P_{ino}-sepF* strain expressing *P_{gntK}-sepF$_{\Delta ML}$-scarlet* (blue), *P_{gntK}-sepF-scarlet* (green), *P_{gntK}-sepF$_{K125/F131A}$-scarlet* (red) in 1% *myo*-inositol or *P_{gntK}* in 0% *myo*-inositol (purple).

Mean values and standard deviations of cell lengths are shown in Supplementary Table 5. The number of cells used in the analyses (n) is indicated below each violin representation. The box indicates the 25th to the 75th percentile and the whiskers indicate the 95% confidence interval. The mean and the median are indicated with a dot and a line in the box, respectively. Source data are provided as a Source Data file.

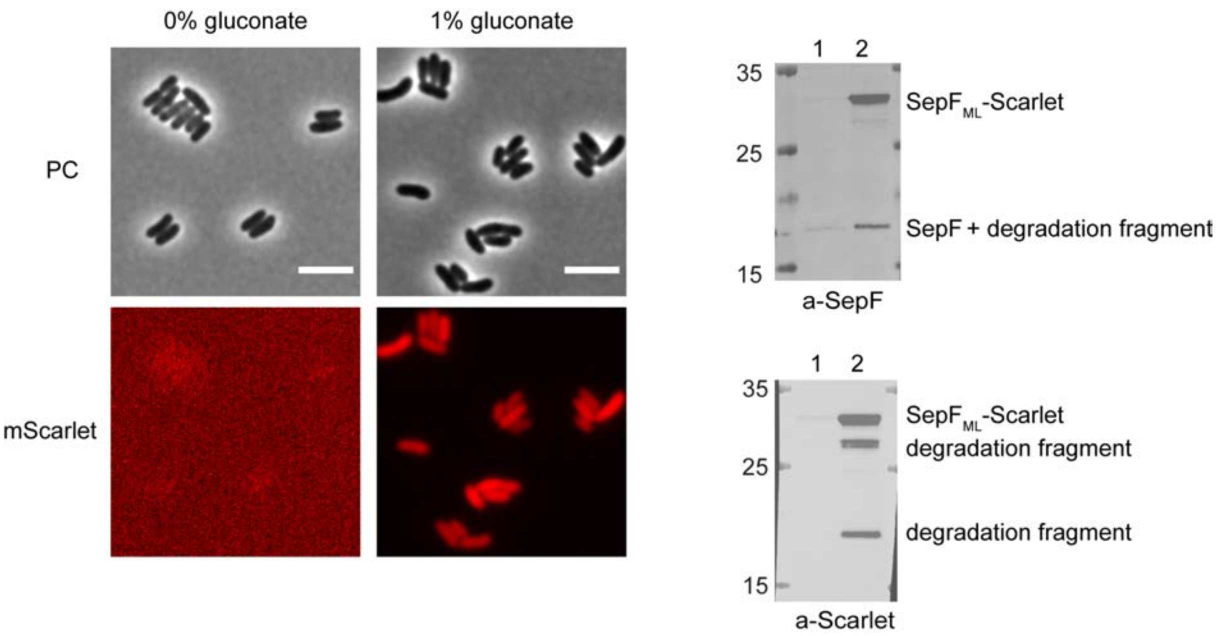

Supplementary Figure 12: Cytosolic localization of the ML-Scarlet fusion protein. Representative images in phase contrast and red fluorescent signal for WT-P_{gntK}-*sepF*$_{ML}$-*scarlet* grown in the absence or presence of 1% gluconate. Cultures were grown in minimal medium + 4% sucrose. Scale bars = 5 μm. The Western blots of cell extracts of both conditions were revealed with the α-SepF and α-Scarlet antibodies. Lane 1: 0% gluconate; Lane 2: 1% gluconate. Molecular weight markers (kDa) are shown on the left side of the blot. The data shown are representative of experiments made independently in triplicate.

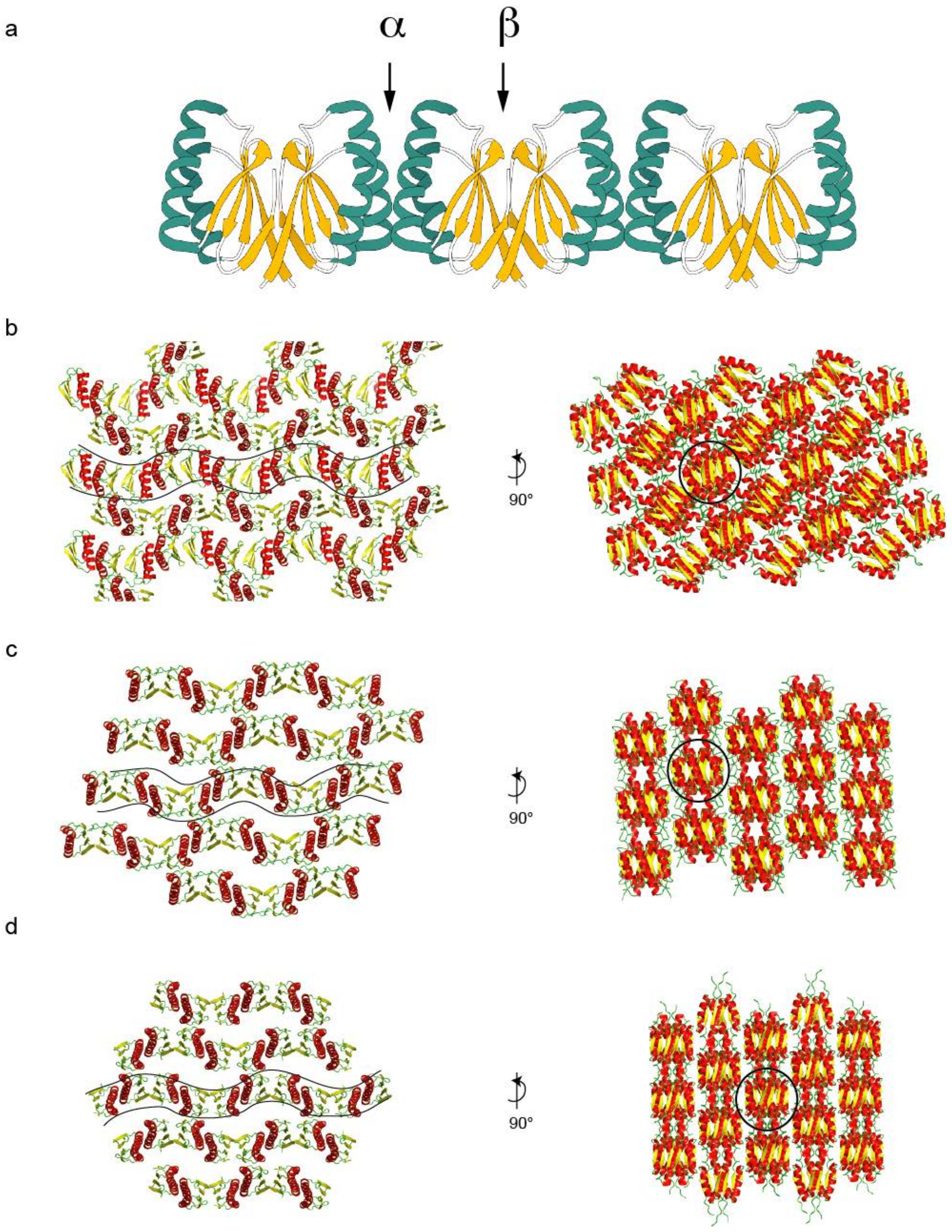

Supplementary Figure 13: Linear polymers of SepF in the crystal state. a. Two possible dimerization interfaces identified in the crystal structure of *B. subtilis* SepF (pdb code 3ZIH), mediated respectively by a four-helix bundle (labelled α) and by the tight association of opposite sheets (labelled β), giving rise to linear polymers of the protein. **b-d.** The crystal structures of different constructs of *C. glutamicum* SepF, all of which lack the C-terminal helix α3, exhibit a similar molecular arrangement. The panels show the crystal lattices of (**b**) $SepF_{\Delta ML,\Delta\alpha 3}$-$FtsZ_{CTD}$, (**c**) $SepF_{\Delta ML,\Delta\alpha 3}$, and (**d**) PDB code 3P04, projected along two perpendicular directions, showing respectively a lateral (left) and a frontal (right) view of the packing of linear SepF polymers. In each projection, a single polymer is highlighted.

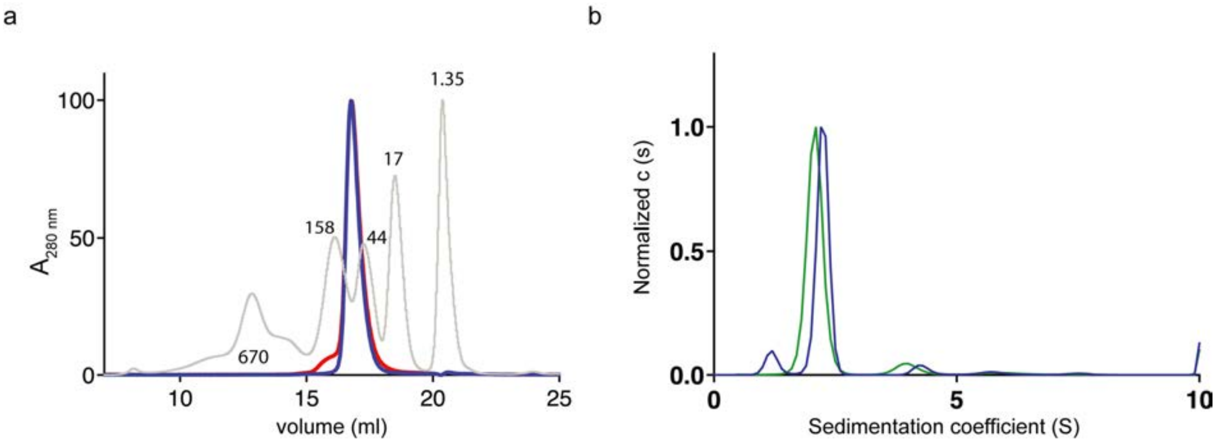

Supplementary Figure 14: Biophysical characterization of the oligomerization state of SepF and SepF$_{\Delta\alpha3}$. a. Size exclusion chromatography of SepF (blue) and SepF$_{\Delta\alpha3}$ (red) on a Superdex S200 10/300 column. The molecular weight (MW) markers are shown in gray and the numbers correspond to the MW in kDa. **b.** Analytical ultracentrifugation profile of SepF and SepF$_{\Delta\alpha3}$ detected by interference. For SepF (blue) the main peak showed an estimated molecular weight of 31.3 kDa with a sedimentation coefficient (S) of 2.239 in agreement with the values expected for a SepF dimer. For SepF$_{\Delta\alpha3}$ (green) the main peak showed an estimated molecular weight of 28.8 kDa with a sedimentation coefficient (S) of 2.079 in agreement with the values expected for a SepF$_{\Delta\alpha3}$ dimer.

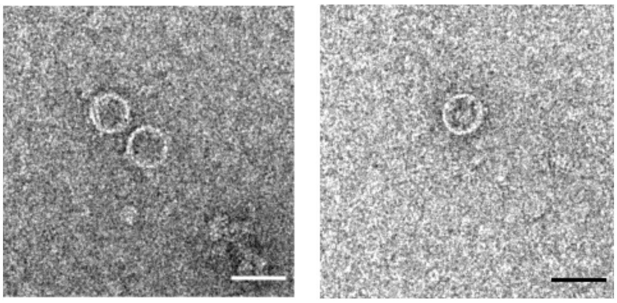

Supplementary Figure 15: Rings of purified negatively stained *Mtb*SepF$_{\Delta ML}$ (50 μM) observed by electron microscopy. The data shown are representative of experiments made independently in triplicate. The scale bar is 50nm.

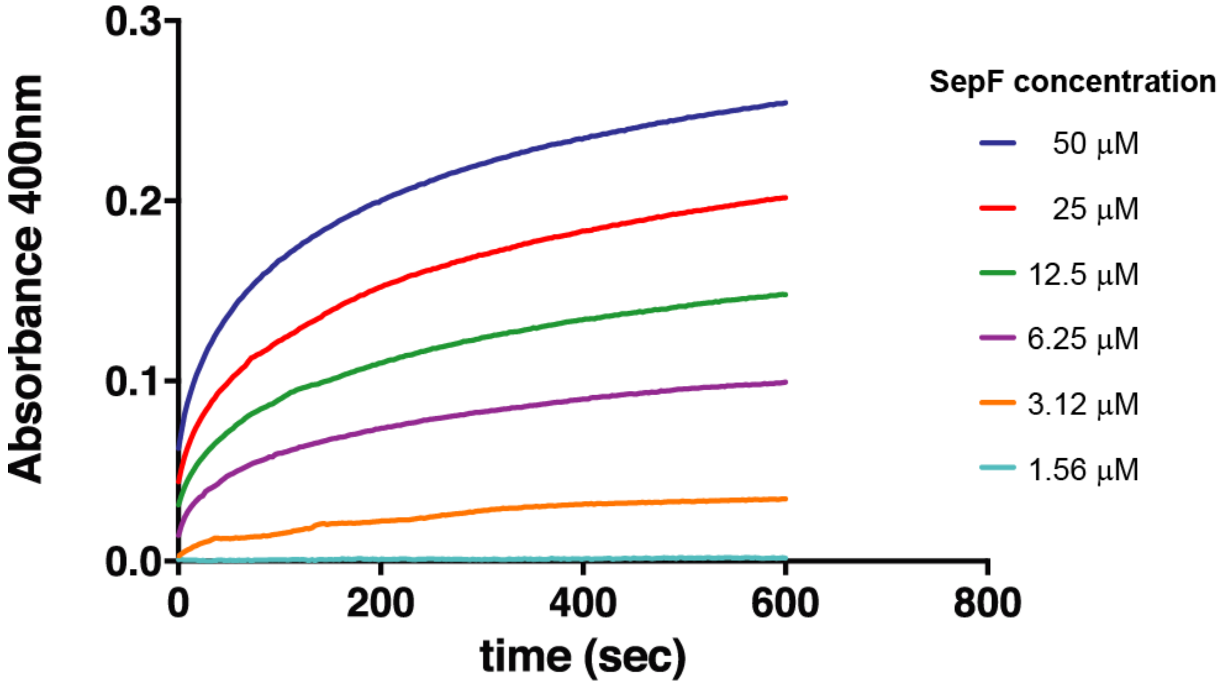

Supplementary Figure 16: Turbidity assays. Polymerization of SepF at the indicated protein concentrations was assessed in the presence of lipid membranes (50 µM SUVs). Source data are provided as a Source Data file.

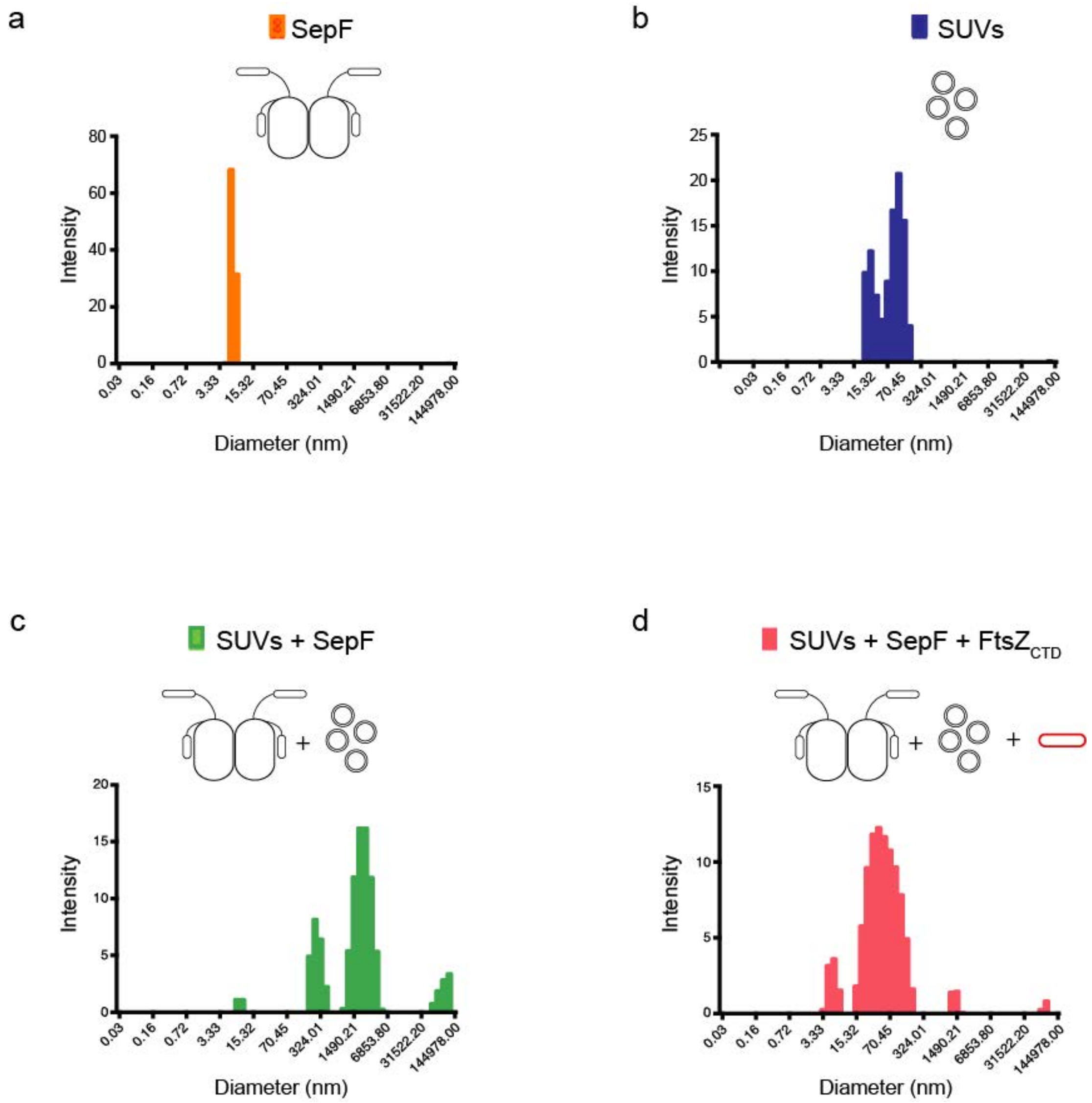

Supplementary Figure 17: SepF-SUV-FtsZ$_{CTD}$ interaction analyzed by dynamic light scattering (DLS). Polydispersity of the samples was measured as intensity. SepF was used at 50 µM and FtsZ$_{CTD}$ peptide at 100 µM. SepF was monodisperse (**a**), while SUVs presented a larger polydispersity with an average diameter around 70 nm (**b**). When SepF and SUVs were incubated together, several peaks corresponding to larger particles were seen (**c**). These peaks were reversed when the FtsZ$_{CTD}$ was added to the SepF samples (**d**).

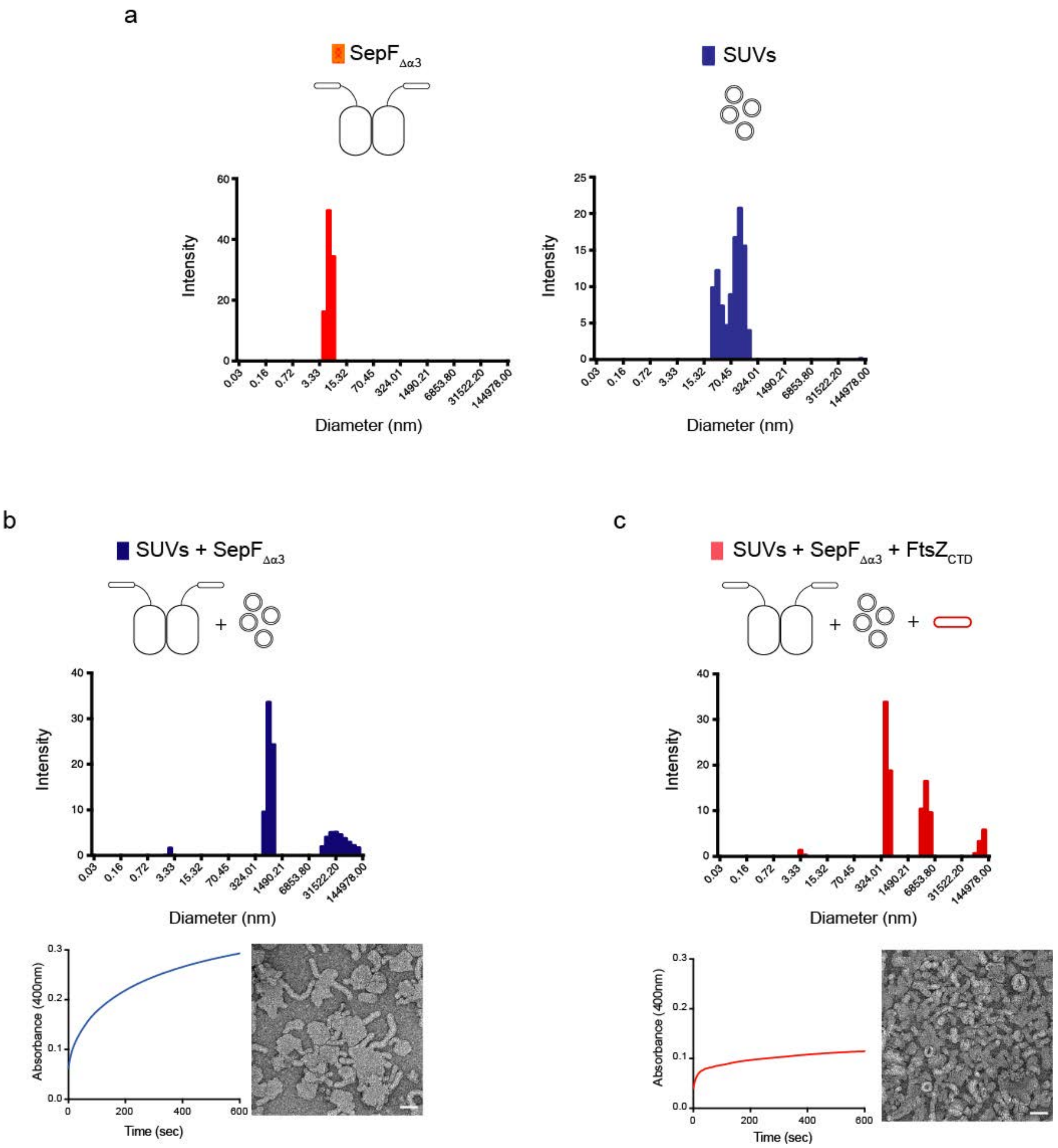

Supplementary Figure 18: SepF$_{\Delta\alpha3}$-SUV-FtsZ$_{CTD}$ interactions. a. In DLS, SepF$_{\Delta\alpha3}$ was monodisperse, while SUVs presented a larger polydispersity with an average diameter around 70 nm. **b.** DLS, polymerization assay and negatively-stained EM images of the end-point of the assay for SepF$_{\Delta\alpha3}$ in the presence of SUVs. **c.** Idem for SepF$_{\Delta\alpha3}$ in the presence of SUVs + FtsZ$_{CTD}$. Incubation of SUVs with SepF$_{\Delta\alpha3}$ led to formation of large particles and vesicle tubulation, but these effects were only partially reversed upon addition of FtsZ$_{CTD}$. Scale bars: 50 nm. The data shown are representative of experiments made independently in triplicate.

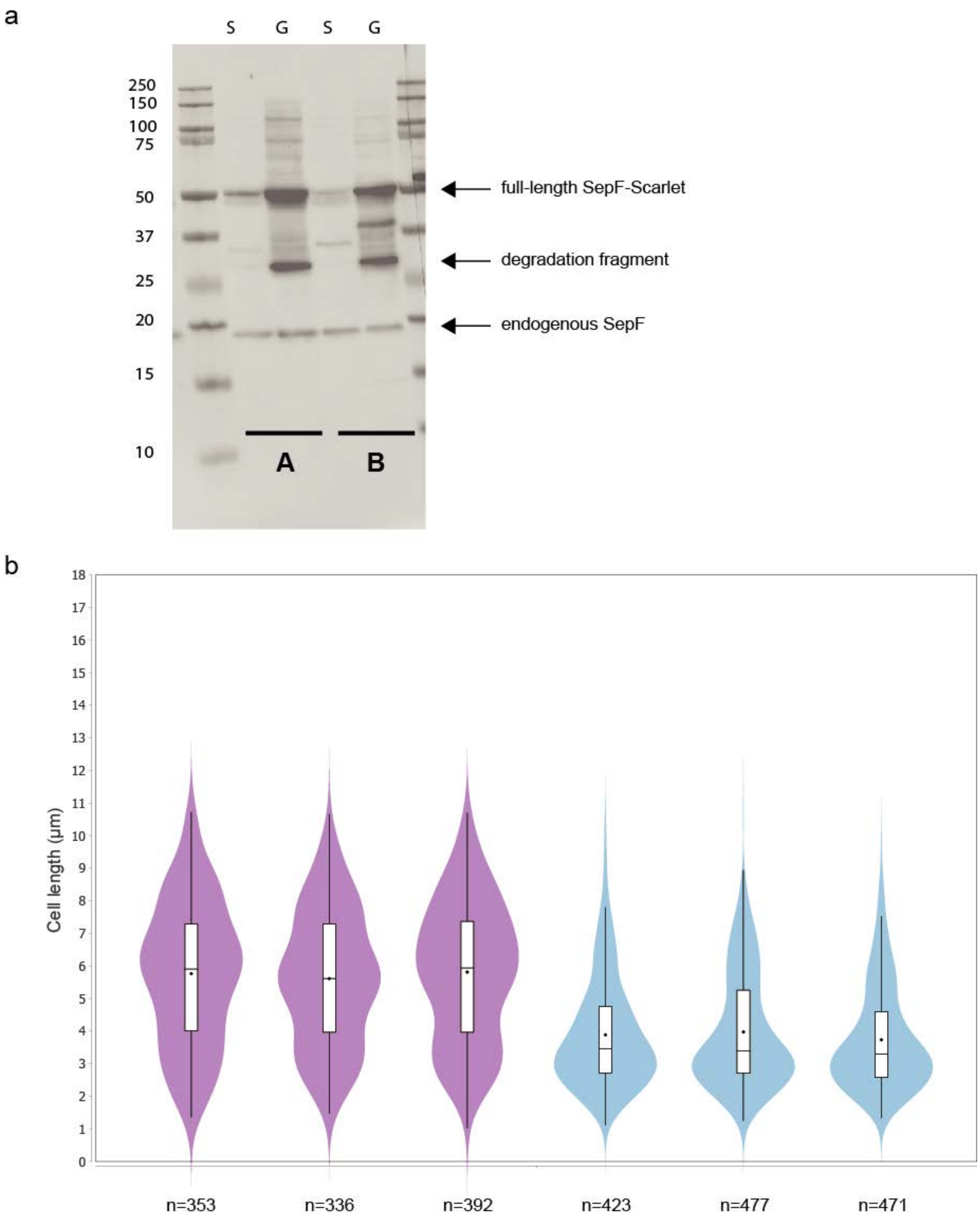

Supplementary Figure 19: Expression and phenotypes of SepF-Scarlet and SepF$_{K125/F131A}$-Scarlet in WT *C. glutamicum.* a. Western blot of whole cell extracts of WT-*P*$_{gntK}$-*sepF*$_{K125/F131A}$-*scarlet* (A) and WT-*P*$_{gntK}$-*sepF-scarlet* (B) grown either in 4% sucrose (S) or 4% sucrose + 1% gluconate (G). Molecular weight markers (kDa) are shown on the left side of the blot. The data shown are representative of experiments made independently in triplicate. **b.** Violin plot of triplicate analysis showing the distribution of cell length at 5 hours of WT-*P*$_{gntK}$-*sepF*$_{K125/F131A}$-*scarlet* (purple) and WT-*P*$_{gntK}$-*sepF-scarlet* (blue). Mean values and standard deviations of cell lengths are shown in Supplementary Table 5. The number of cells used in the analyses (n) is indicated below each violin representation. The box indicates the 25th to the 75th percentile and the whiskers indicate the 95% confidence interval. The mean and the median are indicated with a dot and a line in the box, respectively. Source data are provided as a Source Data file.

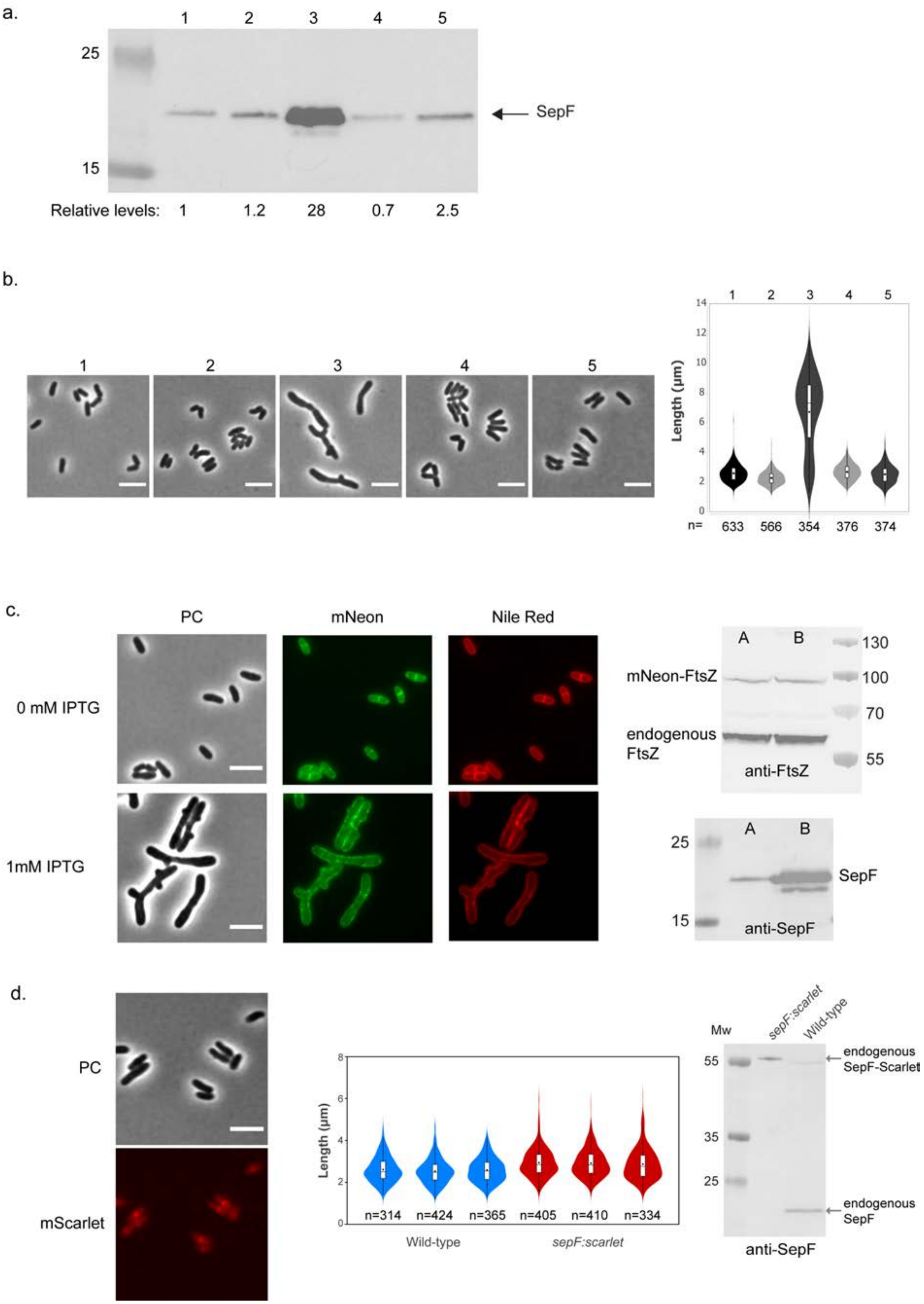

Supplementary Figure 20: Characterization of SepF expression levels and associated phenotypes. a. Western blot of whole cell extracts of WT ATCC13032 (Lane 1), WT-P_{tac}-

sepF in the absence (Lane 2) or presence of 1 mM IPTG (Lane 3), WT-P_{gntK}-*sepF* in the absence (Lane 4) or presence (Lane 5) of 1% gluconate. All cultures were grown in minimal medium + 4% sucrose, supplemented or not with the inducer. The blot was revealed with the (α-SepF) antibody. Molecular weight markers (kDa) are shown on the side of the blot. The relative levels compared to WT level (1) for this blot are shown below each lane. The levels were quantified using Image Lab (Biorad) **b.** Representative images in phase contrast of the cells that were used for the extracts above (same numbering as gel lanes) and violin plots showing the distribution of cell length. The number of cells used in the analyses (n) is indicated below each violin representation. The box indicates the 25th to the 75th percentile and the whiskers indicate the 95% confidence interval. The mean and the median are indicated with a dot and a line in the box, respectively. **c.** Representative images in phase contrast, mNeon and Nile red (membrane stain) fluorescent signals of WT-P_{tac}-*sepF*-P_{gntK}-*FtsZ* strains grown in the absence (A) or presence (B) of 1 mM IPTG, at time point 6h. The Western blots of whole cell extracts of both strains revealed using α-FtsZ and α-SepF antibodies are apposed. Molecular weight markers (kDa) are shown on the side of the blot. **d.** Representative images in phase contrast and red fluorescent signal for the *sepF:scarlet* strain. Violin plots show the distribution of cell lengths of the WT (blue) and *sepF:scarlet* strains (red). The box indicates the 25th to the 75th percentile and the whiskers indicate the 95% confidence interval. The mean and the median are indicated with a dot and a line in the box, respectively. The Western blot of whole cell extracts revealed using an α-SepF antibody shows the replacement of the WT SepF by SepF-Scarlet. Molecular weight markers (kDa) are shown on the side of the blot. Scale bars = 5 μm. The data shown are representative of experiments made independently in triplicate. Source data are provided as a Source Data file.

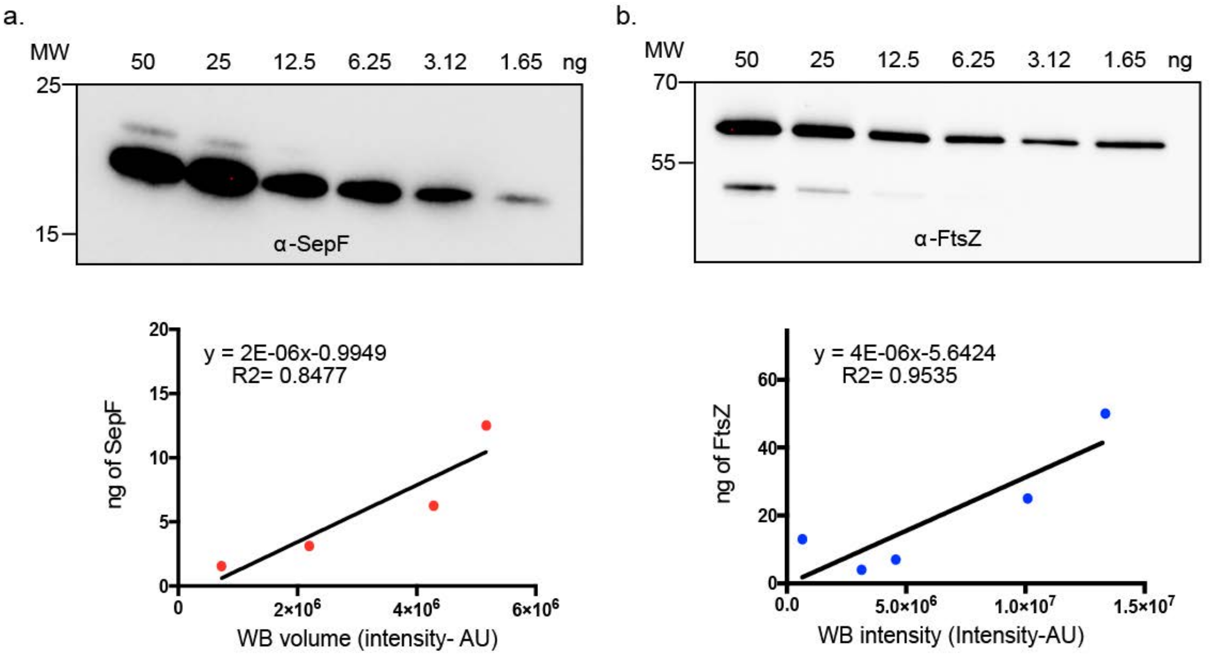

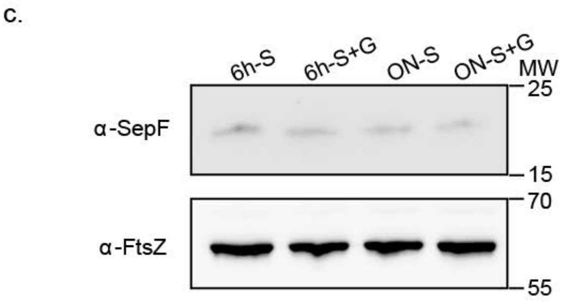

d.

	fmoles FtsZ/µg total extract	fmoles SepF/µg total extract	**FtsZ/SepF ratio**
6h-S	9,38	0,5	**18,76**
ON-S	7,57	0,85	**8,91**
6h-S+G	9,41	0,78	**12,06**
ON-S+G	7,88	0,97	**8,12**

Supplementary Figure 21: Quantification of SepF and FtsZ levels in wild-type ATCC13032 strains. a. Western blot of serial dilutions of recombinant SepF (50 ng to 1.65 ng). The standard curve corresponds to points 12.5 ng to 1.65 ng. Nanograms of recombinant protein were plotted against band volume. **b.** Western blot of serial dilutions of recombinant FtsZ (50 ng to 1.65 ng). The standard curve corresponds to points 50 to 3.12 ng. Nanograms of recombinant protein were plotted against band volume. **c.** Quantification of SepF and FtsZ in 60 µg of *C. glutamicum* ATCC13032 whole cell extracts grown in CGXII media supplemented with sucrose (4%) or sucrose and gluconate (1%). Samples were taken at 6h and over-night (24h). **d.** Total quantification given in fmoles/µg total extract of FtsZ and SepF in the different samples. SepF:FtsZ ratios vary from 8 to 18 depending on the carbon source and growth phase. The data shown are representative for experiments performed at least twice. Molecular weight markers (MW, in kDa) are shown on the side of the blots. Source data are provided as a Source Data file.

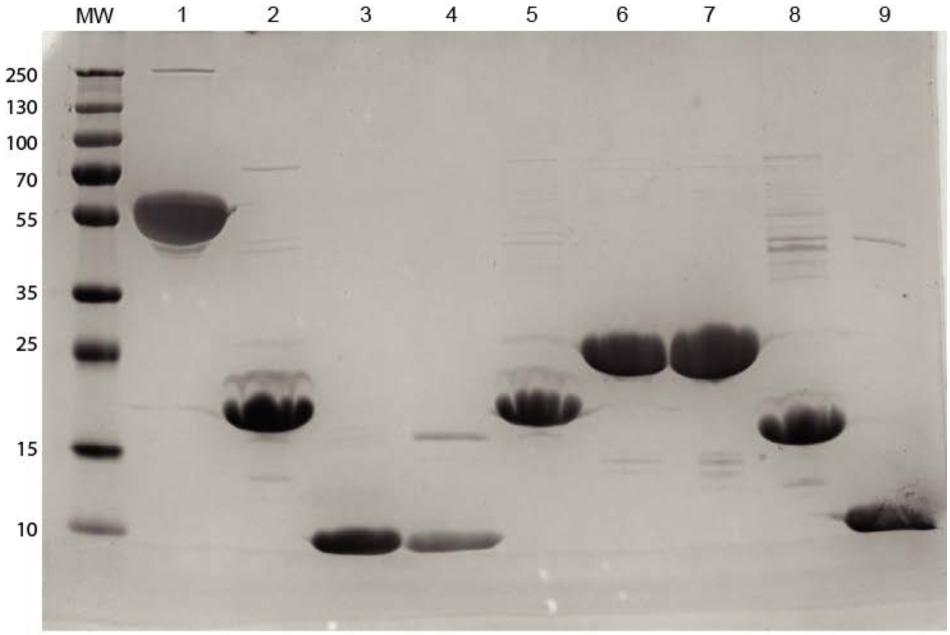

1: FtsZ	47 kDa
2: SepF	17 kDa
3: SepF$_{\Delta ML}$	10 kDa
4: SepF$_{\Delta ML,\Delta\alpha 3}$	8 kDa
5: SepF$_{K125E,F131A}$	17 kDa
6: His6:SUMO-SepF$_{\Delta ML,F131A}$	22 kDa
7: His6:SUMO-SepF$_{\Delta ML,K125E,F131A}$	22 kDa
8: SepF$_{\Delta\alpha 3}$	15 kDa
9: *M. tuberculosis* SepF$_{\Delta ML}$	11 kDa

Supplementary Figure 22: SDS-PAGE of all purified recombinant proteins used in this work. The molecular weight markers are indicated on the left of the gel (in kDa). The protein constructs and calculated molecular weights and for each lane are shown in the figure. The data shown are representative for experiments performed at least twice.

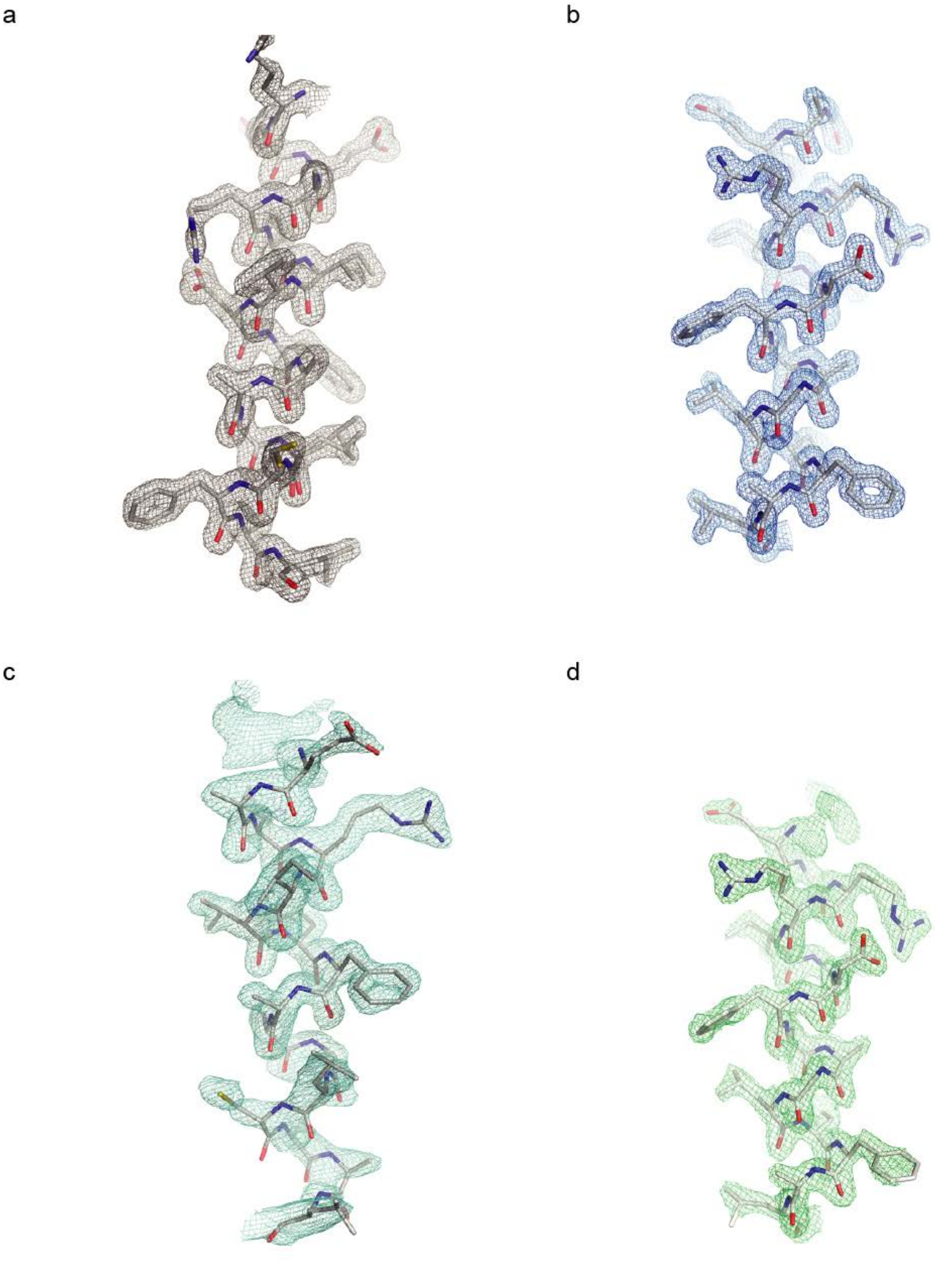

Supplementary Figure 23: Representative electron density maps for each crystal structure of SepF determined in this work. a. $SepF_{\Delta ML}$. **b.** $SepF_{\Delta ML}$ in complex with $FtsZ_{CTD}$. **c.** $SepF_{\Delta ML, \Delta\alpha 3}$. **d.** $SepF_{\Delta ML, \Delta\alpha 3}$ in complex with $FtsZ_{CTD}$. The (2mFobs-DFcalc) maps contoured at 1.5 σ show the same α-helix (residues 102-119) in all structures.

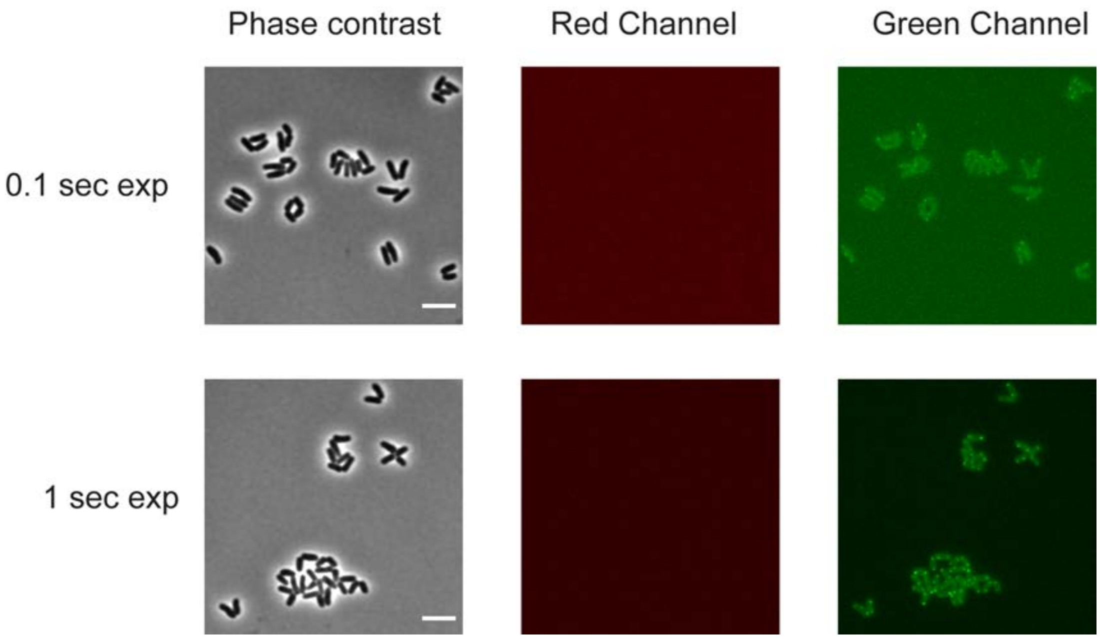

Supplementary Figure 24: Auto fluorescence of *C. glutamicum*. Representative images at exposure times 0.1 and 1 sec, for WT ATCC13032 strain grown in CGXII media supplemented with sucrose (4%) and imaged at OD=6. The data shown are representative of experiments made independently in triplicate.

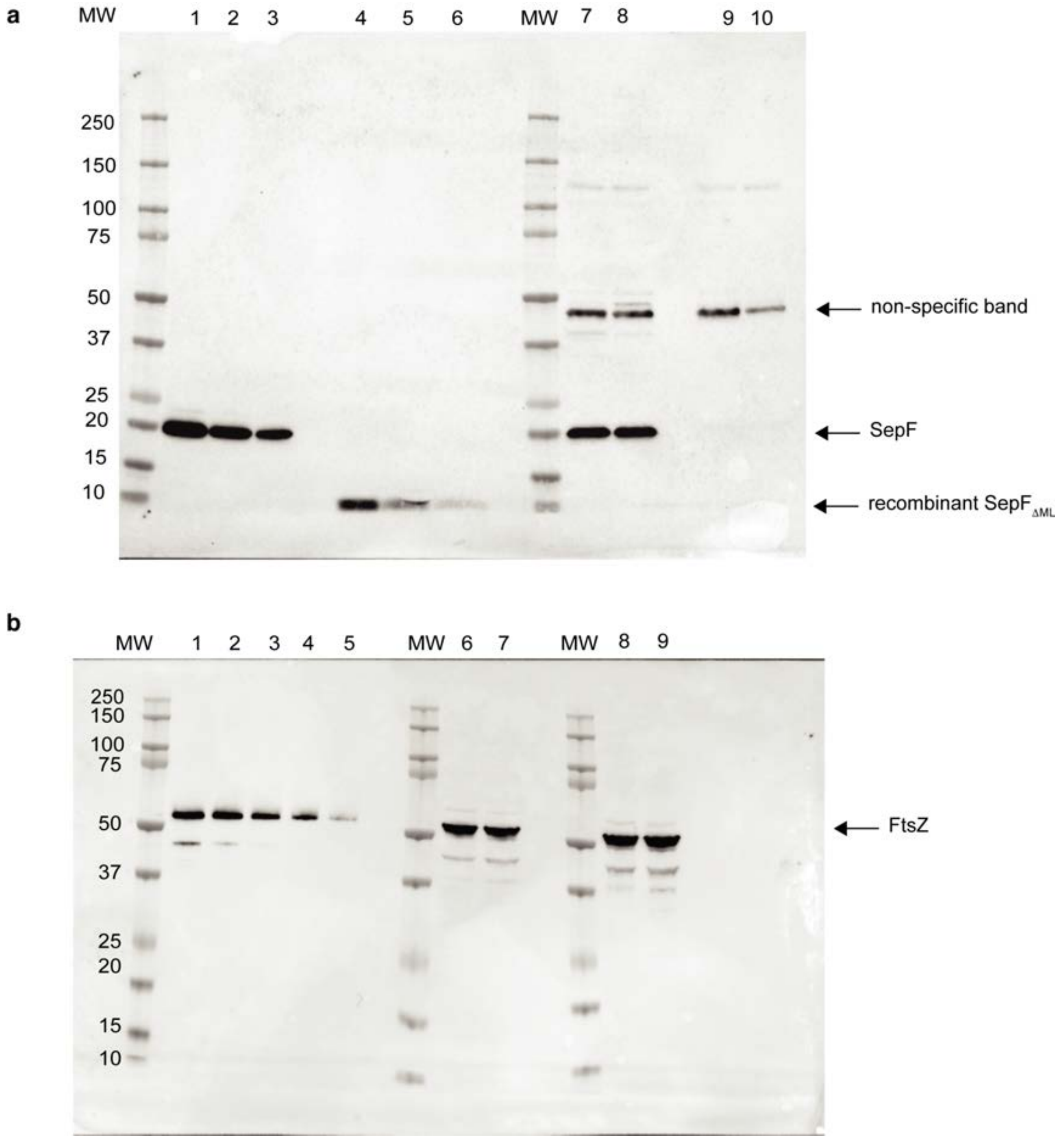

Supplementary Figure 25: Characterization of anti-SepF and anti-FtsZ antibodies. a. Anti-SepF (α-SepF) antibody characterization. Lanes 1-3: Serial dilutions (12.5, 6.25, 3.12 ng) of recombinant SepF against which the antibody was raised. Lanes 4-6: Serial dilutions (12.5, 6.25, 3.12 ng) of recombinant SepF$_{\Delta ML}$. Lanes 7-8: 120 μg of whole cell extracts of *C. glutamicum* WT in exponential (7) or stationary (8) phase. Lanes 9-10: 120 μg of whole cell extracts of *C. glutamicum* *P$_{ino}$-sepF* grown in 1% *myo*-inositol (SepF depletion condition) in exponential (9) or stationary (10) phase. **b.** Anti-FtsZ (α-FtsZ) antibody characterization. Lanes 1-5: Serial dilutions (100, 50, 25, 12.5, 6.25 ng) of recombinant FtsZ against which the antibody was raised. Lanes 6-7: 60 μg of whole cell extracts of *C. glutamicum* WT in exponential (6) or stationary (7) phase. Lanes 8-9: 120 μg of whole cell extracts of *C. glutamicum* WT in exponential (8) or stationary (9) phase. The data shown are representative for experiments performed at least twice. Molecular weight markers (kDa) are shown on the side of the blot.

Figure 1

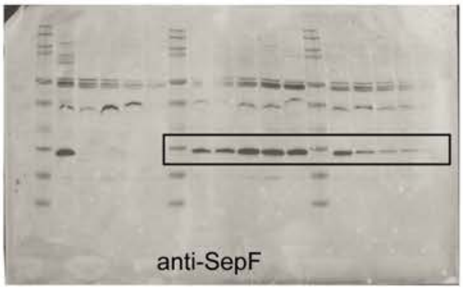

Figure 1

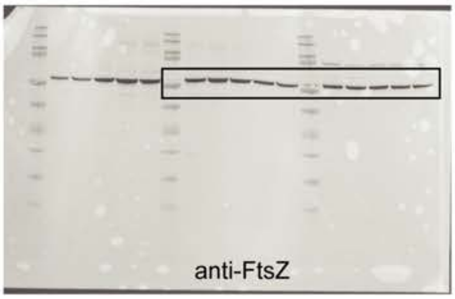

Suppl. Fig. 1

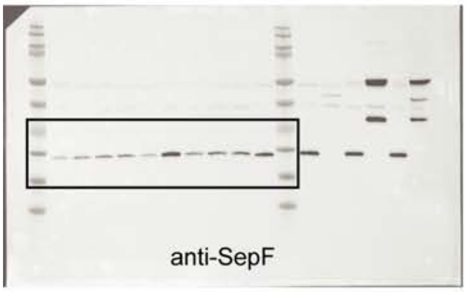

Suppl. Fig. 4b and 20c

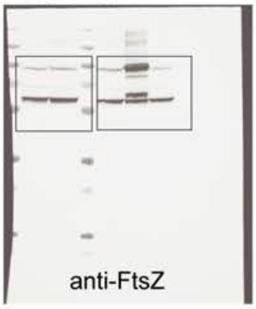

Suppl. Fig. 4b

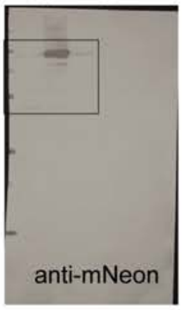

Suppl. Fig. 19

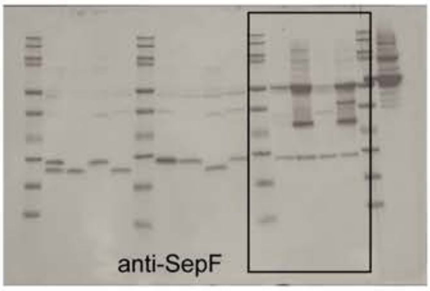

Suppl. Fig. 20 Suppl. Fig. 12

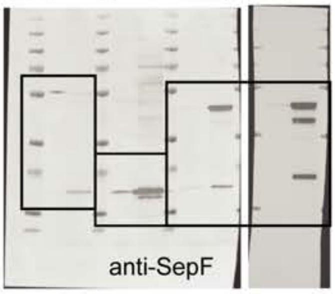

Suppl. Fig. 20a

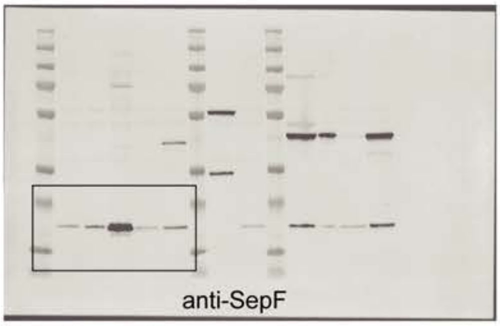

Suppl. Fig. 25

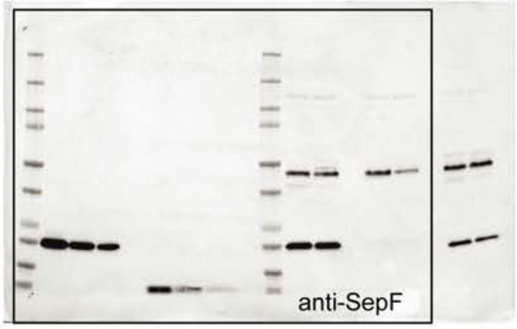

Suppl. Fig. 21 a and c

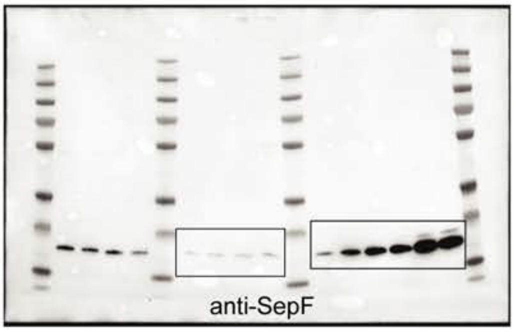

Suppl. Fig. 21 b and c

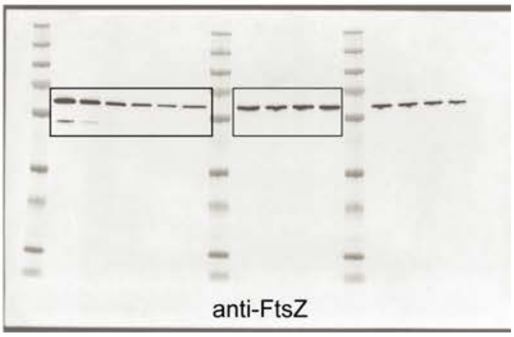

Supplementary Figure 26: Full uncropped Western Blots of all the analyses shown in this work. The boxes correspond to the crops used in the named figures. Note that the anti-SepF Blot corresponding to Supplementary Fig. 21 a and c has been flipped horizontally.

Supplementary Table 1. Crystallographic data.

	$SepF_{\Delta ML}$-$FtsZ_{CTD}$	$SepF_{\Delta ML}$	$SepF_{\Delta ML,\Delta\alpha3}$	$SepF_{\Delta ML,\Delta\alpha3}$-$FtsZ_{CTD}$
**Data collection**				
Space group	$P\ 2_1\ 2_1\ 2_1$	$P\ 2_1\ 2_1\ 2_1$	C 2	P 21
Cell dimensions				
a, b, c (Å)	33.67, 46.08, 98.98	35.32, 53.08, 95.63	65.84, 32.27, 74.41	34.25, 75.53, 52.61
α, β, γ (°)	90, 90, 90	90, 90, 90	90, 114.6, 90	90, 102.71, 90
Resolution (Å)*	49.5–1.6 (1.63–1.6)	46.4–1.8 (1.84–1.8)	32.9–1.5 (1.53–1.5)	42.5–2.2 (2.27–2.2)
R_{sym}	0.071 (0.577)	0.074 (0.551)	0.026 (0.166)	0.140 (0.555)
I/σ(I)	10.3 (1.9)	12.9 (2.5)	19.6 (4.9)	8.8 (3.0)
Completeness (%)	96.9 (98.3)	99.9 (100)	95.2 (97.3)	99.8 (99.9)
Redundancy	3.6 (3.4)	5.2 (5.2)	2.5 (2.1)	6.7 (6.6)
**Refinement**				
Resolution (Å)	1.6	1.8	1.50	2.2
Number of reflections	21381	17290	21918	13296
R-work/R-free	0.208 / 0.230	0.189 / 0.214	0.211 / 0.218	0.206 / 0.258
Number of atoms				
protein	1439	1332	1052	2396
ligands/ions	0	6	0	1
water	148	109	84	115
B-factors ($Å^2$)				
protein	26.4	31.3	39.6	31.6
ligands/ions	-	22.3	-	45.7
water	35.1	43.8	36.7	35.1
RMS deviations				
Bond length (Å)	0.010	0.010	0.010	0.010
Bond angles (°)	0.97	1.02	1.01	1.09
PDB code	**6SAT**	**6SCP**	**6SCQ**	**6SCS**

* Values in parenthesis refer to the highest recorded resolution shell.

Supplementary Table 2. FtsZ protein levels in pull-down analyses of *C. glutamicum* strains expressing SepF-Scarlet or $SepF_{K125/F131A}$ using Scarlet as the bait protein (see Materials and Methods for details). **a**. The tables show the number of peptides and the total signal (XIC) obtained for FtsZ, Scarlet, or SepF-Scarlet constructions in three replicates of the pull-down analyses using either SepF-Scarlet or $SepF_{K125/F131A}$-Scarlet. Peptide spectrum matches from each replicate and each condition were statistically filtered using the Patternlab´s Search Engine Processor (SEPro) module in order to achieve a FDR< 1% at the protein level. Differential proteins among conditions were detected using Patternlab´s TFold module (q-value ≤ 0.05). XIC intensities were obtained using Patternlab for Proteomics XIC quantitation mode. In both cases, the Signal of SepF is estimated as Signal [SepF-Scarlet] – Signal [Scarlet] or Signal [$SepF_{K125/F131A}$-Scarlet] – Signal [Scarlet]. **b**. The table shows the average and standard deviation of the FtsZ normalized signal from IP experiments using SepF-Scarlet and $SepF_{K125/F131A}$-Scarlet. The fold change in normalized FtsZ levels between the two experiments was 7.2 (unpaired t-test, p value 0.004).

a

	**SepF-Scarlet**					
	Replicate 1		Replicate 2		Replicate 3	
**Signal**	**# Peptides**	**Signal (XIC)**	**# Peptides**	**Signal (XIC)**	**# Peptides**	**Signal (XIC)**
FtsZ	25	3.94E+07	30	5.67E+07	24	4.15E+07
SepF-Scarlet	60	3.88E+08	73	9.85E+08	56	3.90E+08
Scarlet	38	2.08E+08	45	4.96E+08	35	1.95E+08
SepF*		1.79E+08		4.89E+08		1.95E+08
FtsZ/SepF		0.219		0.116		0.213

	**SepFK125/F131A-Scarlet**					
	**Replicate 1**		**Replicate 2**		**Replicate 3**	
**Signal**	**# Peptides**	**Signal (XIC)**	**# Peptides**	**Signal (XIC)**	**# Peptides**	**Signal (XIC)**
FtsZ	19	9.20E+06	19	9.89E+06	16	1.30E+07
$SepF_{K125/F131A}$-Scarlet	72	9.20E+08	63	5.67E+08	56	5.57E+08
Scarlet	45	4.07E+08	38	1.59E+08	31	1.78E+08
SepF*		5.13E+08		4.08E+08		3.78E+08
FtsZ/SepF		0.0179		0.0242		0.0345

b

	**Signal FtsZ/SepF average**	**SD**
SepF-Scarlet	0.183	0.058
$SepF_{K125/F131A}$-Scarlet	0.0255	0.0084

Supplementary Table 3. Bacterial strains and plasmids used in this study.

Strain or plasmid	Characteristics	Reference
E. coli		
DH5*α*	F- endA1 Φ80dlacZΔM15 Δ(lacZYA-argF)U169 recA1 relA1 hsdR17(rK– mK+) deoR supE44 thi-1 gyrA96 phoA λ–; strain used for general cloning procedures	Suppl. Ref. 2
BL21(DE)	F- ompT hsdSB(rB–mB–) gal dcm (DE3); host for protein production	Suppl. Ref. 3
CopyCutter EPI400	F- mcrA Δ(mrr-hsdRMS-mcrBC) Φ80dlacZΔM15 ΔlacX74 recA1 endA1 araD139 Δ(ara, leu)7697 galU galK λ- rpsL (StrR) nupG trfA tonA pcnB4 dhfr	Suppl. Ref. 4
C. glutamicum		
ATCC 13032	Biotin-auxotrophic wild type	Suppl. Ref. 5
P_{ino}-sepF	*myo*-inositol dependent *sepF* silencing strain. ATCC 13032 with insertion of a terminator and P_{ino} promoter to silence *sepF* (*cg2363)* expression. Repressible in the presence of *myo*-inositol	This work
sepF:scarlet	*sepF* with an in-frame fusion with scarlet spaced by a linker (LEGSGQGPGSGQGSGH)	This work
**Plasmids**		
pK19mobsacB	KanaR; plasmid for allelic exchange in *C. glutamicum*; (pK18 $oriV_{Ec}$, sacB, lacZα)	Suppl. Ref. 6
pk19-P3323-lcpA	KanaR; pK19mobsacB derivative. Used as a PCR template to amplify a transcriptional terminator and the promoter of cg3323 (P_{ino})	Suppl. Ref. 7
pK19-P_{ino}-sepF	KanaR; pk19mobsacB derivative containing 500 bp upstream-region of *sepF*, a transcriptional terminator, the P_{ino} promoter and 500bp of the *sepF* coding region	This work
pK19-sepF:scarlet	KanaR; pK19mobsacB derivative containing the coding sequence of *sepF* fused to scarlet by a linker and the 500 bp downstream of *sepF*.	This work
pET-SUMO-sepF	KanaR; pET derivate for *C. glutamicum* SepF recombinant expression containing a N-terminal His-tag followed by a SUMO protease cleavage site	This work
pET-SUMO-mtbsepF	KanaR; pET derivate for *M. tuberculosis* SepF recombinant expression containing a N-terminal His-tag followed by a SUMO protease cleavage site	This work
pET-SUMO-ftsZ	KanaR; pET derivate for *C. glutamicum* FtsZ recombinant expression containing an N-terminal His-tag followed by a SUMO protease cleavage site	This work
pET-SUMO-$mtbsepF_{\Delta ML}$	KanaR; pET derivate for *M. tuberculosis* SepF (122-218) recombinant expression containing a N-terminal His-tag followed by a SUMO protease cleavage site	This work
pET-SUMO-$sepF_{\Delta ML}$	KanaR; pET derivate for *C. glutamicum* SepF (63-152) recombinant expression containing a N-terminal His-tag followed by a SUMO protease cleavage site	This work
pET-SUMO-$sepF_{\Delta ML,\Delta\alpha3}$	KanaR; pET derivate for *C. glutamicum* SepF (63-137) recombinant expression containing a N-terminal His-tag followed by a SUMO protease cleavage site	This work
pET-SUMO-$sepF_{\Delta\alpha3}$	KanaR; pET derivate for *C. glutamicum* SepF (1-137) recombinant expression containing a N-terminal His-tag followed by a SUMO protease cleavage site	This work

pET-SUMO-sepF$_{\Delta ML,F131A}$	KanaR; pET derivate for *C. glutamicum* SepF (63-152) recombinant expression containing a N-terminal His-tag followed by a SUMO protease cleavage site. Carrying amino acid substitution F131A	This work
pET-SUMO-sepF$_{\Delta ML,K125/F131A}$	KanaR; pET derivate for *C. glutamicum* SepF (63-152) recombinant expression containing a N-terminal His-tag followed by a SUMO protease cleavage site. Carrying double amino acid substitution K125E and F131A	This work
pET-SUMO-sepF$_{K125/F131A}$	KanaR; pET derivate for *C. glutamicum* SepF recombinant expression containing a N-terminal His-tag followed by a SUMO protease cleavage site. Carrying double amino acid substitution K125E and F131A	This work
pTGR5	KanaR; *E. coli/C. glutamicum* shuttle vector for regulated gene expression under control of tac promoter (P_{tac} lacI ColE1 $oriV_{Ec}$ pGA1 $oriV_{Cg}$)	Suppl. Ref. 8
pCLTON1	KanaR; *E. coli* / *C. glutamicum* shuttle expression vector with the *B. subtilis* derived P_{tet} promoter from pWH105 and the tetR gene under control of *C. glutamicum P_{gap}* promoter from pJC1-pgap-tetR.	Suppl. Ref. 9
pMA-mscarlet-I	AmpR; pMA vector (GeneArt, Thermo Fisher Scientific) containing a synthetic gene coding for mScarlet-I, codon optimized for expression in *C. glutamicum*	This work
pMA-mneongreen	AmpR; pMA vector (GeneArt, Thermo Fisher Scientific) containing a synthetic gene coding for mNeonGreen, codon optimized for expression in *C. glutamicum*	This work
pUMS3	KanaR; pTGR5 derivative in which P_{tac} was exchanged by P_{gntK} promoter	This work
pUMS3-scarlet	KanaR; pUMS3 derivative for expression of Scarlet under control of P_{gntK} promoter	This work
pUMS3-sepF-scarlet	KanaR; pUMS3 derivative for expression of cgSepF-Scarlet under control of P_{gntK} promoter	This work
pUMS3-sepF$_{K125/F131A}$-scarlet	KanaR; pUMS3 derivative for expression of cgSepF$_{K125/F131A}$-Scarlet under control of P_{gntK} promoter	This work
pUMS3-sepF$_{\Delta ML}$-scarlet	KanaR; pUMS3 derivative for expression of cgSepF$_{\Delta ML}$-Scarlet under control of P_{gntK} promoter	This work
pUMS3-mneon-ftsZ	KanaR; pUMS3 derivative for expression of mNeon-cgFtsZ under control of P_{gntK} promoter	This work
pTGR5-cgSepF	KanaR; pTGR5 derivative for expression of cgSepF under control of P_{tac} promoter	This work
pUMS3-cgSepF	KanaR; pUMS3 derivative for expression of cgSepF under control of P_{gntK} promoter	This work
pUMS3-cgSepF1-62-Scarlet	KanaR; pUMS3 derivative for expression of cgSepF1-62-Scarlet under control of P_{gntK} promoter	This work
pTGR5-cgSepF/FtsZ-mNeon	KanaR; pUMS3 derivative for co-expression of cgSepF and mNeon-FtsZ under control of the P_{tac} and *PgntK* promoters, respectively.	This work.
pUMS40	KanaR; pTGR5 derivative in which P_{tac} was exchanged by P_{tet} promoter	This work
pUMS40-sepF	KanaR; pUMS40 derivative for expression of cgSepF under control of P_{tet} promoter	This work
pUMS40-sepF$_{\Delta\alpha 3}$	KanaR; pUMS40 derivative for expression of cgSepF$_{\Delta\alpha 3}$ under control of P_{tet} promoter	This work
pUMS40-sepF-scarlet	KanaR; pUMS40 derivative for expression of cgSepF-Scarlet under control of P_{tet} promoter	This work

Supplementary Table 4: Oligonucleotides used in this study

**Oligonucleotide**	**Sequence (5' → 3') and properties**[a]
**Construction of pET-SUMO-cgSepF$_{\Delta ML}$**	
P1	AGTTATCAGTCCACCATTGTTCCGGTT
P2	GGTGGACTGATAACTCATGCCACCAATCTG
**Construction of pET-SUMO-cgSepF$_{\Delta ML,\Delta\alpha3}$ & pET-SUMO-cgSepF$_{\Delta\alpha3}$**	
P13	GGAATAGTCTAACATTAGCACGTCT
P14	AGACGTGCTAATGTTAGACTATTCC
**Construction of pET-SUMO-cgSepF$_{\Delta ML,F131A}$**	
P39	TTACCGCCGCCGTCGTG
P40	GATAGCGTTACCGCCGCCGT
**Construction of pET-SUMO-cgSepF$_{\Delta ML,K125/F131A}$**	
P33	TAAAATGCAGGAAATCGATAGCG
P34	AACGCTATCGATTTCCTGCATTTTAC
**Construction of pET-SUMO-mtbSepF$_{\Delta ML}$**	
P7	GATGGTCACCCGC
P8	CGGGTGACCATCGCCACCAATCTGTTC
**Construction of pk19-*P$_{ino}$-sepF* and the respective mutant strain**	
P123	**TGAAGGGAAACTGCC**ATAAAACGAAAGGCTCAGTCGAAAGAC
P124	**CTTGAGCATGGACAT**CTAAAATTTCTCCTCTTAAAAAGATAACGGCC
P121	**TGTTGTGTGGAATTG**TACAAGAGTTTGGTGTCACCGC
P122	GGCAGTTTCCCTTCACCTG
P125	ATGTCCATGCTCAAGAAGACTAAAGAA
P126	**AATTGTTATCCGCTCA**AGACCTTGGAAAAGAGCCGTAGAGG
**Construction of pk19-*sepF:scarlet***	
p150	**TGTTGTGTGGAATTG**ATGTCCATGCTCAAGAAGACTAA
p172	**ACCACGAGGGTGTGT**TTACTTGTACAGTTCATCCATGC
p173	ACACACCCTCGTGGTGT
p174	**AATTGTTATCCGCTCA**TCGAAAGGATGAAAAAGAG
**Primers for colony PCR used during the construction of the *sepF:scarlet* strain**	
p86	CTTGACTGCAGCCATGGTTT
p74	TTTACACTTTATGCTTCCGG
p141	GGGAAACTGCCATGTCCATGC
p142	CTCATCGCTGTAGTGACCCGGTA
**Primers for colony PCR used during the construction of the *P$_{ino}$-sepF* strain**	
P86	CTTGACTGCAGCCATGGTTT
P70	TGTTGTGTGGAATTGCCAAAGCACTCGAACTTCC
P142	CTCATCGCTGTAGTGACCCGGTA
P85	GATTACCGTCGTGATGAGCG

Construction of pUMS3

OligoMM_157	CTCACGCACTCCGGGTATCTAGATGACATACGAACAAATCGTTGATCTAGTT
OligoMM_158	GTTCGGTGAGGTCATATGGTCTTATCCTTTCTTTGGTGGCGTCTC

Construction of pUMS3-cgSepF-Scarlet

OligoMM_178	**ATGGTCTTATCCTTTCTTTG**GTGGC
OligoMM_179	**AGCGGCCGCTTAAGGTAC**
OligoMM_183	**CAAAGAAAGGATAAGACCAT**ATGTCCATGCTCAAGAAGACTAAAGAATTCTTCGGACTCG
OligoMM_184	**GCCAGATCCCTCGAGGCGGATG**CGTGCGGCGCG
OligoMM_180	**ACGCATCCGCCTCGAGGGATCTGGC**CAGGGACCGGGCTCAGGCCAAGGAAGCGGCCATATGGTGTCCAAGGGCGAAG
OligoMM_181	**CGGTACCTTAAGCGGCCGCT**TTACTTGTACAGTTCATCCATGCC

Construction of pUMS3-cgSepF$_{K125/F131A}$-Scarlet

OligoMM_188	AGATTGACAGCGTCACCGCCGCTGTCGTTCCAGAGCTGTCCAACATCAGCAC
OligoMM_189	GCGGTGACGCTGTCAATCTCCTGCATCTTGCCACGCAATGCGAAGCACAG

Construction of pUMS3-cgSepF$_{\Delta ML}$-Scarlet

OligoMM_178	**ATGGTCTTATCCTTTCTTTG**GTGGC
OligoMM_179	**AGCGGCCGCTTAAGGTAC**
OligoMM_187	**CAAAGAAAGGATAAGACCAT**ATGTCTTACCAGTCCACCATCGTTCCAGTAGAGCTTCATTC
OligoMM_184	**GCCAGATCCCTCGAGGCGGATGCGTG**CGGCGCG
OligoMM_180	**ACGCATCCGCCTCGAGGGATCTGGC**CAGGGACCGGGCTCAGGCCAAGGAAGCGGCCATATGGTGTCCAAGGGCGAAG
OligoMM_181	**CGGTACCTTAAGCGGCCGCT**TTACTTGTACAGTTCATCCATGCC

Construction of pUMS3-mNeon-cgFtsZ

OligoMM_178	**ATGGTCTTATCCTTTCTTTGGT**GGC
OligoMM_179	**AGCGGCCGCTTAAGGTAC**
OligoMM_190	**CAAAGAAAGGATAAGACCAT**ATGGTGTCCAAGGGCGAAG
OligoMM_191	**GCCAGATCCCTCGAGCTTGTACAGT**TCATCCATGCCC
OligoMM_192	**ACTGTACAAGCTCGAGGGATCTGGC**CAGGGACCGGGCTCAGGCCAAGGAAGCGGCATGACCTCACCGAACAAC
OligoMM_193	**CGGTACCTTAAGCGGCCGCT**TTACTGGAGGAAGCTGGG

Construction of pTGR5-cgSepF

OligoMM_179	AGCGGCCGCTTAAGGTAC
OligoMM_301	ATGTAAAAAATCCTTTCGCTAGC
OligoMM_302	**GGATAACAATTTGCTAGCGAAAGGATTTTTTACAT**ATGTCCATGCTCAAGAAGACTAAAGAATTCTTCGGACTC
OligoMM_303	**TCGGTACCTTAAGCGGCCGCTTTA**GCGGATGCGTGCGGCGCGCTCGAGCTCGGAAGT
OligoMM_304	TAAAGCGGCCGCTTAAGGTACCGAATTCTAAGCTTCACCACCACCACCACCACTGA
OligoMM_305	GTACCTTAAGCGGCCGCTTTAGCGGATGCGTGCGGCGCGCTCGA

Construction of pUMS3-cgSepF$_{\Delta FC}$-Scarlet

OligoMM_311	CAGCACCTCGCCTCGAGGGATCTGGCC
OligoMM_312	CCTCGAGGCGAGGTGCTGGCGATGGAG

Construction of pUMS40

OligoMM_254	**AATATGCGGCCGCATATATG**GATC
OligoMM_227	**ATGGTGAGCAAGGGCGAGGAG**

OligoMM_252	**CATATATGCGGCCGCATATT**CCTGCCGCCAGCGGGCGTACAAAAGTG
OligoMM_253	**TGAACAGCTCCTCGCCCTTGCTCACCAT**AGTGTATCAACAAGCTGGGGATCTTAAGCTTG
**Construction of pUMS40-cgSepF**	
OligoMM_202	ATCCGCTAACTCGAGGGATCTGGCCAGGGACCGGG
OligoMM_203	CTCGAGTTAGCGGATGCGTGCGGCGCGCTCGAGC
**Construction of pUMS40-cgSepF**$_{\Delta\alpha3}$	
OligoMM_204	CCAGAGTAACTCGAGGGATCTGGCCAGGGACC
OligoMM_205	CTCGAGTTACTCTGGAACGACAGCGAAGGTGACGC
**Construction of pUMS40-cgSepF-Scarlet**	
OligoMM_258	**AGTGTATCAACAAGCTGGGGA**
OligoMM_179	**AGCGGCCGCTTAAGGTAC**
OligoMM_259	**TCCCCAGCTTGTTGATACACT**ATGTCCATGCTCAAGAAGACTAAAGAATTCTTCGGACTCG
OligoMM_184	**GCCAGATCCCTCGAGGCGGATGCGT**GCGGCGCG
OligoMM_180	**ACGCATCCGCCTCGAGGGATCTGGC**CAGGGACCGGGCTCAGGCCAAGGAAGCGGCCATAT GGTGTCCAAGGGCGAAG
OligoMM_181	**CGGTACCTTAAGCGGCCGCT**TTACTTGTACAGTTCATCCATGCC

[a] Overlaps for Gibson assembly are written in bold letters. Restriction sites are underlined.

Supplementary Table 5. Statistical analysis of cell lengths in all violin plots.

Strain + construct	Time point	Replicate	N cells	Mean length	Standard dev.
**Fig. 1**					
WT	t = 0	1	348	3,10	0,63
	t = 3	1	362	3,30	0,71
	t = 6	1	314	2,63	0,61
P_{ino}-*sepF*	t = 0	3	413	2,40	0,49
	t = 3	3	339	4,11	0,90
	t = 6	3	318	7,19	1,56
**Fig. 3**					
P_{ino}-*sepF* - P_{gntK}-*sepF*$_{\Delta ML}$-*scarlet*	t = 6	1	302	9,81	2,24
P_{ino}-*sepF* - P_{gntK}-*sepF-scarlet*	t = 6	1	379	5,38	1,62
P_{ino}-*sepF* - P_{gntK}-*sepF*$_{K125/F131A}$-*scarlet*	t = 6	1	328	9,96	2,85
P_{ino}-*sepF* - empty vector	t = 6	1	466	2,61	0,57
**Supplementary Fig. 1**					
WT - empty vector	t = 4.5	1	369	2,61	0,56
		2	329	2,58	0,57
		3	372	2,57	0,58
P_{ino}-*sepF* - P_{tet}-*sepF*	t = 4.5	1	351	2,61	0,58
		2	406	2,61	0,54
		3	436	2,68	0,57
**Supplementary Fig. 2**					
WT	t = 0	1	348	3,10	0,63
		2	413	3,04	0,65
		3	337	3,11	0,68
	t = 3	1	362	3,30	0,71
		2	293	3,18	0,72
		3	338	3,20	0,69
	t = 6	1	314	2,63	0,61
		2	424	2,55	0,60
		3	365	2,61	0,58
P_{ino}-*sepF*	t = 0	1	349	2,54	0,51
		2	298	2,66	0,51
		3	413	2,40	0,49
	t = 3	1	242	4,30	0,89
		2	321	4,19	1,00

		3	339	4,11	0,90
	t = 6	1	307	7,71	1,44
		2	314	7,60	1,84
		3	318	7,19	1,56
		**Supplementary Fig. 4**			
WT - P_{gntK}-*mneon-ftsZ*	t = 3	1	383	2,52	0,56
		2	398	2,44	0,56
		3	429	2,51	0,53
WT - empty vector	t = 4.5	1	369	2,61	0,56
		2	329	2,58	0,57
		3	372	2,57	0,58
		**Supplementary Fig. 5**			
P_{ino}-*sepF* - P_{gntK}-*mneon-ftsZ*	t = 0	1	251	2,85	0,64
		2	398	2,99	0,64
		3	337	2,88	0,71
	t = 3	1	326	5,59	1,37
		2	350	5,59	1,31
		3	317	5,74	1,17
	t = 6	1	295	9,98	2,31
		2	352	9,99	1,98
		3	297	9,90	1,83
		**Supplementary Fig. 11**			
P_{ino}-*sepF* - P_{gntK}-*sepF*$_{\Delta ML}$-*scarlet*	t = 6	1	302	9,81	2,24
		2	313	9,33	1,97
		3	377	9,46	2,07
P_{ino}-*sepF* - P_{gntK}-*sepF-scarlet*	t = 6	1	379	5,38	1,62
		2	345	4,97	1,63
		3	360	4,60	1,64
P_{ino}-*sepF* - P_{gntK}-*sepF*$_{K125/F131A}$-*scarlet*	t = 6	1	328	9,96	2,85
		2	324	9,74	2,24
		3	342	9,52	2,20
P_{ino}-*sepF* - empty vector	t = 6	1	466	2,61	0,57
		2	375	2,52	0,55
		3	359	2,58	0,61
		**Supplementary Fig. 19**			
WT - P_{gntK}-*sepF*$_{K125/F131A}$-*scarlet*	t = 5	1	353	5,76	2,18
		2	336	5,61	2,05
		3	392	5,81	2,13

		1	423	3,88	1,66
WT - P_{gntK}-*sepF-scarlet*	t = 5	2	477	3,97	1,73
		3	471	3,73	1,58

Supplementary Table 6. Two-sided p values derived from a Mann-Whitney test for data shown in Supplementary Figures 2b and 5a.

		Population 1	Population 2	p-value	-LOG (p-value)
**SUPPLEMENTARY FIGURE 2b**					
Comparison of replicates	t = 0	1	2	0,005113817	2,3
		1	3	1,68E-04	3,8
		2	3	1,05E-10	10,0
	t = 3	1	2	0,172945821	0,8
		1	3	0,012939594	1,9
		2	3	0,304985897	0,5
	t =6	1	2	0,289172209	0,5
		1	3	2,50E-06	5,6
		2	3	0,001222835	2,9
Comparison of time points	replicate 1	t =0	t = 3	5,28E-80	79,3
		t =0	t = 6	1,72E-107	106,8
		t = 3	t = 6	3,84E-84	83,4
	replicate 2	t =0	t = 3	3,86E-71	70,4
		t =0	t = 6	1,04E-100	100,0
		t = 3	t = 6	1,56E-88	87,8
	replicate 3	t =0	t = 3	7,92E-95	94,1
		t =0	t = 6	4,99E-119	118,3
		t = 3	t = 6	3,81E-88	87,4

		Population 1	Population 2	p-value	-LOG (p-value)
**SUPPLEMENTARY FIGURE 5a**					
Comparison of replicates	t = 0	1	2	0,00497791	2,3
		1	3	0,761101817	0,1
		2	3	0,01263391	1,9
	t = 3	1	2	0,874416442	0,1
		1	3	0,376425603	0,4
		2	3	0,240334469	0,6
	t =6	1	2	0,459123206	0,3
		1	3	0,1578877	0,8
		2	3	0,397598602	0,4
Comparison of time points	replicate 1	t =0	t = 3	1,19E-78	77,9
		t =0	t = 6	5,60E-86	85,3
		t = 3	t = 6	3,95E-83	82,4
	replicate 2	t =0	t = 3	3,08E-102	101,5
		t =0	t = 6	2,72E-120	119,6
		t = 3	t = 6	1,25E-104	103,9
	replicate 3	t =0	t = 3	5,86E-100	99,2
		t =0	t = 6	9,60E-104	103,0
		t = 3	t = 6	1,95E-92	91,7

Supplementary References

1. Laskowski, R. A. & Swindells, M. B. LigPlot+: Multiple ligand-protein interaction diagrams for drug discovery. *J. Chem. Inf. Model* **51**, 2778-2786 (2011).
2. Hanahan, D. Studies on transformation of *Escherichia coli* with plasmids. *J. Mol. Biol.* **166,** 557–580 (1983).
3. Studier, F. W. & Moffatt, B. A. Use of bacteriophage T7 RNA polymerase to direct selective high-level expression of cloned genes. *J. Mol. Biol.* **189,** 113–130 (1986).
4. Haskins, D. *Epicentre Forum* **11**, 6 (2004).
5. Kinoshita, S. & Udaka, S. Studies on the amino acid fermentation. *J. Gen. Appl. Microbiol.* **3,** 193–205 (1957).
6. Schäfer, A. *et al.* Small mobilizable multi-purpose cloning vectors derived from the *Escherichia coli* plasmids pK18 and pK19: selection of defined deletions in the chromosome of *Corynebacterium glutamicum*. *Gene* **145,** 69–73 (1994).
7. Baumgart, M., Schubert, K., Bramkamp, M. & Frunzke, J. Impact of LytR-CpsA-Psr Proteins on Cell Wall Biosynthesis in *Corynebacterium glutamicum*. *J. Bacteriol.* **198,** 3045–3059 (2016).
8. Ravasi, P., Peiru, S., Gramajo, H. & Menzella, H. G. Design and testing of a synthetic biology framework for genetic engineering of *Corynebacterium glutamicum*. *Microb. Cell Fact.* **11,** 147 (2012).
9. Lausberg, F., Chattopadhyay, A. R., Heyer, A., Eggeling, L. & Freudl, R. A tetracycline inducible expression vector for *Corynebacterium glutamicum* allowing tightly regulable gene expression. *Plasmid* **68,** 142–147 (2012).
